# A Schizophrenia-Related Genetic-Brain-Cognition Pathway Revealed in a Large Chinese Population

**DOI:** 10.1016/j.ebiom.2018.10.009

**Published:** 2018-10-16

**Authors:** Na Luo, Jing Sui, Jiayu Chen, Fuquan Zhang, Lin Tian, Dongdong Lin, Ming Song, Vince D. Calhoun, Yue Cui, Victor M. Vergara, Fanfan Zheng, Jingyu Liu, Zhenyi Yang, Nianming Zuo, Lingzhong Fan, Kaibin Xu, Shengfeng Liu, Jian Li, Yong Xu, Sha Liu, Luxian Lv, Jun Chen, Yunchun Chen, Hua Guo, Peng Li, Lin Lu, Ping Wan, Huaning Wang, Huiling Wang, Hao Yan, Jun Yan, Yongfeng Yang, Hongxing Zhang, Dai Zhang, Tianzi Jiang

**Affiliations:** a Brainnetome Center and National Laboratory of Pattern Recognition, Chinese Academy of Sciences, Institute of Automation, Beijing 100190, China; b University of Chinese Academy of Sciences, Beijing 100049, China; c The Mind Research Network & LBERI, Albuquerque, NM 87106, USA; d Wuxi Mental Health Center, Wuxi 214000, China; e Department of Psychiatry, First Clinical Medical College, First Hospital of Shanxi Medical University, Taiyuan 030000, China; f Department of Psychiatry, Henan Mental Hospital, The Second Affiliated Hospital of Xinxiang Medical University, Xinxiang 453002, China; g Henan Key Lab of Biological Psychiatry, Xinxiang Medical University, Xinxiang 453002, China,; h Department of Radiology, Renmin Hospital of Wuhan University, Wuhan 430060, China; i Department of Psychiatry, Xijing Hospital, The Fourth Military Medical University, Xi'an 710032, China; j Zhumadian Psychiatric Hospital, Zhumadian 463000, China; k Institute of Mental Health, Peking University Sixth Hospital, Beijing 100191, China; l Key Laboratory of Mental Health, Ministry of Health, Peking University, Beijing 100191, China; m Department of Psychology, Xinxiang Medical University, Xinxiang 453002, China; n Center for Life Sciences, PKU-IDG, McGovern Institute for Brain Research, Peking University, Beijing 100871, China; o Key Laboratory for NeuroInformation of Ministry of Education, School of Life Science and Technology, University of Electronic Science and Technology of China, Chengdu 610054, China; p Queensland Brain Institute, University of Queensland, Brisbane, QLD 4072, Australia; q Department of Electrical and Computer Engineer, The University of New, Albuquerque, NM 87131, USA; r CAS Center for Excellence in Brain Science and Intelligence Technology, Chinese Academy of Sciences, Institute of Automation, Beijing 100190, China

**Keywords:** Schizophrenia, Multimodal fusion, Genetic-brain-cognition pathway, Working memory, Mediation analysis

## Abstract

**Background:**

In the past decades, substantial effort has been made to explore the genetic influence on brain structural/functional abnormalities in schizophrenia, as well as cognitive impairments. In this work, we aimed to extend previous studies to explore the internal mediation pathway among genetic factor, brain features and cognitive scores in a large Chinese dataset.

**Methods:**

Gray matter (GM) volume, fractional amplitude of low-frequency fluctuations (fALFF), and 4522 schizophrenia-susceptible single nucleotide polymorphisms (SNP) from 905 Chinese subjects were jointly analyzed, to investigate the multimodal association. Based on the identified imaging-genetic pattern, correlations with cognition and mediation analysis were then conducted to reveal the potential mediation pathways.

**Findings:**

One linked imaging-genetic pattern was identified to be group discriminative, which was also associated with working memory performance. Particularly, GM reduction in thalamus, putamen and bilateral temporal gyrus in schizophrenia was associated with fALFF decrease in medial prefrontal cortex, both were also associated with genetic factors enriched in neuron development, synapse organization and axon pathways, highlighting genes including *CSMD1*, *CNTNAP2*, *DCC*, *GABBR2* etc. This linked pattern was also replicated in an independent cohort (166 subjects), which although showed certain age and clinical differences with the discovery cohort. A further mediation analysis suggested that GM alterations significantly mediated the association from SNP to fALFF, while fALFF mediated the association from SNP and GM to working memory performance.

**Interpretation:**

This study has not only verified the impaired imaging-genetic association in schizophrenia, but also initially revealed a potential genetic-brain-cognition mediation pathway, indicating that polygenic risk factors could exert impact on phenotypic measures from brain structure to function, thus could further affect cognition in schizophrenia.

**Research in context**

**Evidence before this study**

Schizophrenia is a severe psychiatric disorder demonstrating a strong genetic component with heritability and more and more GWAS analyses towards schizophrenia have been reported. To examine if these genetic schizophrenia-risk variants may affect brain structure, function or even cognitive performance, an effective strategy is to assess the genetic associations with brain-imaging phenotypes as well as behavioral measures; then, based on the identified linked factors, mediation analysis can be conducted to better delineate cognition behaviors among them.

**Added value of this study**

This study is the first attempt to jointly analyze brain fALFF, GM data in conjunction with PGC's reported SNPs in a large Chinese Han population (905 + 166 subjects) using a multivariate, data-driven manner. Gray matter volume reductions in thalamus, putamen and bilateral temporal gyrus in schizophrenia were associated with fALFF decrease in medial prefrontal cortex. Both these imaging impairments were associated with genetic factors enriched in neuron development, synapse organization and axon pathways, highlighting genes including *CSMD1, CNTNAP2, DCC, GABBR2* etc. The linked imaging-genetic pattern was replicated in an independent Chinese cohort and was also associated with working memory performance. Further mediation analyses suggested a “$\mathrm{SNP}\overset{\to \mathrm{GM}\to }{\to }\text{fALFF}\to \text{cognition}$” pathway.

**Implications of all the available evidence**

This study indicates that brain GM and fALFF abnormalities are significantly correlated with the schizophrenia-susceptible SNPs, and polygenic risk factors could exert impact on phenotypic measures from brain structure to function, thus may further lead to cognitive impairment in schizophrenia.

Alt-text: Unlabelled Box

## 1 Introduction

Schizophrenia (SZ) is a severe psychiatric disorder demonstrating a strong genetic component with heritability estimated up to 80% based on family and twin studies [1]. A landmark study from Psychiatric Genomics Consortium (PGC) has reported a multi-stage schizophrenia genome wide association studies (GWAS) study of 36,989 cases and 113,075 controls [2]. To examine if these genetic schizophrenia-risk variants may affect brain structure, function or even cognitive performance, an effective strategy is to assess the genetic associations with brain-imaging phenotypes as well as behavioral measures; then, based on the identified linked factors, mediation analysis can be conducted to better delineate cognition behaviors among them.

In this study, we focus on two imaging features, i.e., gray matter (GM) and fractional amplitude of low frequency fluctuations (fALFF), which are proven to be heritable in previous imaging-genetic analyses. Specifically, GM volume was validated to be one heritable measure as reported in [3,4]. Schizophrenia-candidate single nucleotide polymorphisms (SNPs), like *DISC1* and *ANK3*, have been revealed to be associated with reduced GM in SZ on hippocampus, prefrontal cortex and temporal regions [[5], [6], [7]]. Moreover, abnormalities of GM derived from structural magnetic resonance imaging (MRI) data, especially in the prefrontal cortex and temporal gyrus, have been well documented in schizophrenia patients [[8], [9], [10], [11]]. And the first-degree SZ relatives were shown to share regional brain volume abnormalities with their sick siblings in amygdata-hippocampal complex, thalamus, and the temporal cortices [[12], [13], [14]]. FALFF is another important neuroimaging metric to measure spontaneous fluctuations of BOLD functional MRI (fMRI) signal intensity in the resting state [15,16]. A multi-site fMRI indicated that SZ patients have lower fALFF than healthy controls (HCs) in all regions [17]; and the unaffected SZ siblings were also found with lower fALFF than HCs in the left inferior temporal gyrus [18]. Additionally, a recent report from Neuron, which studied the relationship between gene expression and fALFF, has also found many significant genes correlated with fALFF than expected by chance [19]. Currently, fALFF has been widely used to study schizophrenia in single modality analyses [[20], [21], [22]], multimodal biomarkers analyses [23,24,83] and individualized prediction [25].

In parallel, cognitive impairments are well documented in schizophrenia and there is a notion that genetic effects on cognitive abilities are mediated by brain functions. For example, prefrontal brain region has been revealed to mediate the effects of *COMT* gene on cognitive control [26] . Moreover, previous studies have revealed that heritability for default-mode functional connectivity was 0.42 ± 0.17 [27], while the heritability for gray matter volume in the voxel-based representation was 0.67 ± 0.10 [3], suggesting that structural features have a stronger association with genetic factors than function. Bullmore et al. also provided evidences that the modularity of structural networks can determine or predict the hierarchical organization of functional networks [28]. All these evidences together suggest that genetic factors may impact brain function through affecting structure, while brain function further influences the cognition.

Collectively, we aimed to use a multimodal fusion method [29,30] to explore the linked imaging phenotypes that are particularly associated with the schizophrenia-susceptible SNPs selected from PGC's GWAS results, including GM volume and fALFF, and further to examine the potential mediation effects among brain imaging, genetics and cognitive performance. We hypothesized that 1) brain structural and functional abnormalities would be significantly correlated with the schizophrenia-susceptible SNPs; 2) SNPs could impact brain function through affecting structure, while brain function further mediated the correlation between SNPs and cognition.

## 2 Materials and methods

### 2.1 Participants

The discovery dataset (cohort 1) consisted of 450 SZs and 455 HCs from Chinese Han population (Table 1), which were recruited from seven sites with compatible MRI scanning parameters and imaging quality (Table S1). The independent replication dataset (cohort 2) included 79 SZs and 87 matched HCs collected from Wuxi Mental Health Center (Table 1). This study has been approved by relevant ethical committees. All HCs were recruited by advertisement from the same geographical areas as the patients, with no current or past axis I or II disorders (DSM-IV-TR) as screened by the SCID-Non-Patient Version. Additional exclusion criteria for all subjects included current or past neurological illness, substance abuse or dependence, pregnancy, and prior electroconvulsive therapy or head injury resulting in loss of consciousness. Inclusion criterion for schizophrenia patients was based on the Structured Clinical Interview for DSM-IV-TR Disorders. Out of 450 SZs in cohort 1, medication information was recorded for 243 patients, while the medication information of the other 207 subjects was unknown. Stable antipsychotic treatment on the patients was not required at the moment of MRI scanning. But almost all the patients were in acute episode and used single medicine. Written informed consents were obtained from all study participants, and included permission to share de-identified data between the centers. We used two sample *t*-test to measure the difference of age, duration of illness, PANSS scores and medication in controls and patients between cohort 1 and cohort 2. Chi-square test was adopted to compute the cross-cohort difference of gender as shown in Table 1.

**Table 1:** Demographic characteristics of the overall sample used for analysis.

	Demographics		HC	SZ	p-value
No. of subjects	Discovery data Cohort1		455	450	
		PKUH6	98	76	**0.083**⁎
		HLG	51	60	
		HMS	78	66	
		HMG	64	59	
		XJ	37	52	
		RWU	60	49	
		ZMD	67	88	
	Replication data Cohort2	WUXI	87	79	
Gender	Cohort1	F/M	232/223	218/232	0.39
	Cohort2	F/M	40/47	36/43	0.96
	**pp-value**		**0.67**	**0.65**	
Age (y)	Cohort1	Mean ± SD	28.7 ± 6.92	27.8 ± 6.93	0.05
	Cohort2	Mean ± SD	39.90 ± 14.8	40.2 ± 12.8	0.14
	**pp-value**		**1.49e-09**	**2.22e-16**	
Chlorpromazine Equivalent	Cohort1	Mean ± SD	NA	411.1 ± 205.4^a^	
	Cohort2	Mean ± SD	NA	670.96 ± 367.19^b^	
	**pp-value**		**NA**	**8.24e-6**	
Duration of illness	Cohort1	Mean ± SD	NA	4.09 ± 4.41	
	Cohort2	Mean ± SD	NA	14.73 ± 11.64	
	**pp-value**		**NA**	**2.11e-11**	
PANSS positive	Cohort1	Mean ± SD	NA	24.12 ± 4.13	
	Cohort2	Mean ± SD	NA	21.97 ± 5.37	
	**pp-value**		**NA**	**0.0012**	
PANSS negative	Cohort1	Mean ± SD	NA	20.21 ± 6.14	
	Cohort2	Mean ± SD	NA	22.96 ± 4.47	
	**pp-value**		**NA**	**6.27e-6**	
PANSS general	Cohort1	Mean ± SD	NA	39.56 ± 7.06	
	Cohort2	Mean ± SD	NA	43.18 ± 6.25	
	**pp-value**		**NA**	**1.42e-5**	
PANSS total	Cohort1	Mean ± SD	NA	83.66 ± 12.94	
	Cohort2	Mean ± SD	NA	87.00 ± 14.34	
	**pp-value**			**0.05**	

Note: Chlorpromazine equivalent = Chlorpromazine total (standardized current dose of antipsychotic medication). The p-value represents the result of chi-square test for gender and two sample t-test for age. * represents the ANOVA results of comparing the distribution of HC and SZ along the seven sites. The pp-value indicates the difference of gender, age, chlopromazine equivalent, duration of illness and PANSS scores in HC or SZ between cohort 1 and cohort 2. F: female; M: male; NA: not applicable; PKUH6: Peking University sixth Hospital; HLG: Huilongguan Hospital; HMS: Henan Mental Hospital Siemens scanning site; HMG: Henan Mental Hospital GE scanning site; XJ: Xijing Hospital; ZMD: Zhumadian Psychiatric Hospital; RWU: Renmin Hospital of Wuhan University; WX: Wuxi Mental Health Center.

a Medication information was only collected from 243 out of 450 patients.

b Medication information was collected from 56 out of 79 patients. The other 23 patients are drug-naive patients.

### 2.2 Data collection

To control the discrepancy of imaging quality and performance across seven sites of corhot 1, we repeatedly scanned three healthy subjects by the scanners from all sites. Then, we evaluated the performance of imaging system by calculating signal-to-noise ratio (SNR) of the MRI system and coils in each site. Based on these SNRs, we optimized and identified the final scan parameters. Furthermore, as differences may exist in different hardware and software systems, we set the MRI sequences and parameters by following the two principles: [1] kept the spatial resolution consistent as much as possible across different sites; [2] made the SNR in each site reach its optimum.

#### 2.2.1 fMRI

Subjects were instructed to keep their eyes open during the scan and stare passively at a foveally presented fixation cross, as this is suggested to facilitate network delineation compared to eyes-closed conditions and helps ensure that subjects are awake. Resting state data were collected with single-shot full k-space echo-planar imaging (EPI) with ramp sampling correction using the inter commissural line (AC/PC) (anterior commissure/posterior commissure) as a reference, and details of the machine type and scanning parameters for each site in cohort 1 and cohort 2 were listed in Table S1.

#### 2.2.2 sMRI

The scanning parameters for each site were listed in Table S1.

#### 2.2.3 SNP

We collected ethylene diamine tetraacetic acid (EDTA) anti-coagulated venous blood samples from all the participants and then extracted genomic Deoxyribonucleic acid (DNA) from their whole blood using the EZgene Blood gDNA Miniprep Kit. The whole-genome genotyping was performed on Illumina Human OmniZhongHua-8 BeadChips using the standard Illumina genotyping protocol (Illumina) spanning 900,015 SNP loci. For cohort 2, DNA was extracted from peripheral blood cell according to the standard protocol by protease K digestion, phenol-chloroform extraction and ethanol precipitation. The whole-genome genotyping was performed on Illumina human PsychArray-24 spanning 571,054 loci.

Cognition assessment: Symptomatic assessment was performed using the Positive and Negative Syndrome Scale (PANSS) [31]. Digital span task (including digit forward [DF] and digit backward [DB] scores) is regarded as a mainstay in psychological assessment and is frequently used to study working memory in both healthy and diseased populations [32]. The digit forward (DF) span task began with a series of three digits orally presented to each participant continuing to a maximum of twelve digits. There were two trials per digit series. Participants were asked to point to the correct order of digits on a written 1–9 digit list provided on individual note cards or verbally repeat the numbers if the participant was able to do so. Note cards were placed on the table in front of all participants; however, the response mode was selected by the participant. All subjects began with the first item (three digits), if repeated correctly, the participant continued to the next item, otherwise performed the second trial at the same item. The task was discontinued when the participant failed in the second trial at a digit series. The span was defined as the maximum number of digits repeated by the participant. Digits were presented at 1 per second. The digit backward (DB) task followed the same procedure as described with the DF task, except that digit sequences began with a series of two digits to a maximum of ten digits. All sequences were orally presented to the participants. The participants then verbally repeated the numbers or pointed to the numbers written on individual note cards in reverse order. A criterion for maximum level of DB was the same as for DF. All participants completed a DF span task followed by a DB span task. Details of PANSS scores and working memory scores for each site in cohort 1 are listed in Table S2.

### 2.3 Data statement

The multimodal data used in the present study can be accessed upon request to the corresponding authors.

### 2.4 Data preprocessing

**Fig. 1:**
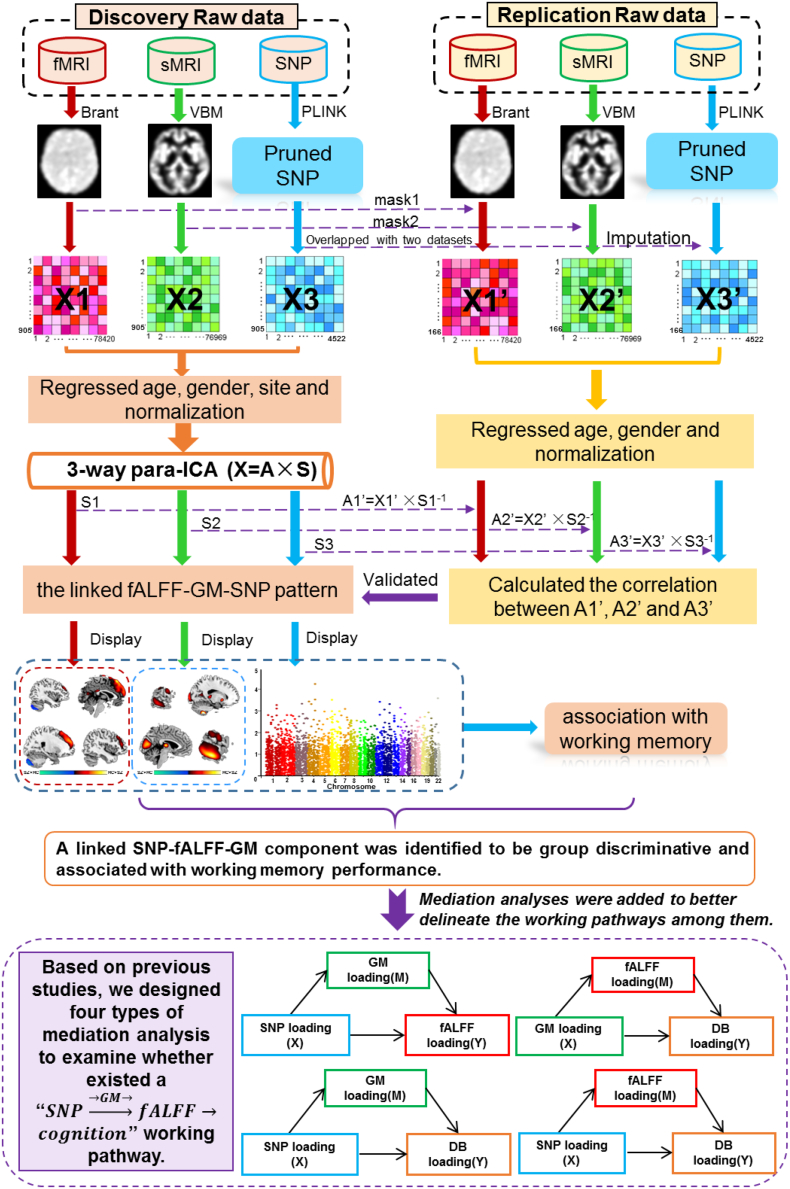
Schematic illustration of the whole analysis. The fALFF, GM and genetic data were preprocessed through a standard quality control procedures by using Brant, SPM and PLINK separately. After preprocessing to get the feature matrices, age, gender and sites were regressed out from each modality and normalized. Once a significantly correlated SNP-fALFF-GM pattern was identified, the spatial maps of them were projected to an independent dataset for replication. After that, we assessed linear associations between working memory performance (DF and DB scores) and para-ICA-derived genetic and phenotype components, controlling for diagnosis (group) and medication effects. In order to better delineate the working pathways among the identified component and working memory performance, we further conducted four types of mediation analysis based on the hypotheses collected from previous studies.

sMRI data preprocessing. The T1-weighted sMRI data were preprocessed by Statistical Parametric Mapping 8 (SPM8, www.fil.ion.ucl.ac.uk/spm/software/spm8) using voxel based morphometry (VBM), with a unified model of image registration, bias correction, tissue classification and spatial normalization to the standard Montreal Neurological Institute (MNI) space [33]. Modulated normalized parameters were used to segment the brain into white matter (WM), gray matter (GM) and cerebral spinal fluid probabilistic maps. The resulting GM images consisted of voxelwise gray matter volumes were resliced to 3 × 3 × 3  mm^3^, resulting in 53 × 63 × 46 voxels and smoothed with an 8 mm Gaussian model [34]. A mask was generated to include only voxels inside the brain across all the subjects for each modality as our previous study [23]. We applied the same preprocessing steps to cohort 2 as in cohort 1. In order to make the spatial maps comparable between two cohorts, we applied the mask computed from cohort 1 to cohort 2, generating feature matrices of the same length (see Fig. 1).

fMRI data preprocessing. Brant software package (http://brant.brainnetome.org/en/latest/) was employed to preprocess the collected fMRI data. The first 10 volumes of each functional time series were discarded for the magnetization equilibrium. Slice timing was performed with the middle slice as the reference frame. Then images were realigned using INRIalign, a motion correction algorithm that is unbiased by local signal changes, resulting in head motion parameters computed by estimating translational and rotational parameters. Each subject had a maximum displacement in a dataset that didn't exceed ±1.5 mm or  ±1.5°. Then data were spatially normalized into Montreal Neurological Institute (MNI) space [33], resliced to 3 × 3 × 3  mm^3^. Denoising were further performed to regress out motion parameters, white matter, and cerebrospinal fluid. Voxel-wise fractional amplitude of low-frequency fluctuations (fALFF) were extracted to generate a map for each subject and spatially smoothed with an 8-mm isotropic Gaussian kernel. A mask was generated to include only voxels inside the brain across all subjects for each modality as our previous study [23]. We applied the same preprocessing steps to cohort 2 as in cohort 1. In order to make the spatial maps comparable between two cohorts, we applied the mask computed from cohort 1 to cohort 2, generating feature matrices of the same length (see Fig. 1).

#### 2.4.1 Quality control of genetic data

Genotype data (spanning 900,015 SNP loci in the cohort 1) were preprocessed using PLINK software following a series of standard quality control procedures [35,36]. Poorly genotyped individuals were removed by checking for discordant sex, elevated missing rate (>3%), unusual heterozygosity (>3 SD from mean) and unusual similarity or relatedness. Individual markers were further removed if marker missing rate was >5%, rare variants with minor allele frequency < 0.01 and SNP correlated above 0.5 in block sizes of 50. Additional exclusion criteria included failure of Hard Weinberg equilibrium (p < 0.00001), case-control genotype call rate difference (p < 0.00001), resulting in the final cohort 1 spanning 318,637 SNPs. Discrete numbers were assigned to the categorical genotypes: 0 for no minor allele, 1 for one minor allele, and 2 for two minor alleles. Afterwards, a Q-Q plot [37] for *p*-values of SZ patients versus HCs tested against a uniform distribution showed no clear indication of population structure (Fig. S1).

#### 2.4.2 Imputation

As for cohort 2, the ungenotyped SNPs were imputed using SHAPEIT v2 (r790) [38] and IMPUTE2 with the 1000 Genomes Phase 1 reference dataset. Further analyses focused on autosomal SNPs with imputation quality scores >0.8 [39]. >4 million SNPs were remained after quality control (QC) procedures. Afterwards, we examined the PGC's SCZ2 genome-wide association study (GWAS) (https://www.med.unc.edu/pgc/results-and-downloads), and selected the SNPs from the GWAS results with a *p*-value lower than 0.01. Then we identified the overlap of the three SNP datasets (PGC's SNPs with *p* < 0.01, preprocessed SNP data in cohort 1, and preprocessed SNP data in cohort 2), resulting in 4522 SNPs. Note that we have conducted imputation only on cohort 2, but not cohort 1. The SNPs were not selected by their odds ratios with schizophrenia but only the *p*-value from PGC according to our previous studies [40,41].

The analysis flowchart is shown in Fig. 1. After preprocessing, the three-dimensional brain image of each subject was reshaped into a one-dimensional vector and stacked, forming a matrix (N_subj_ × N_voxel_) for each of the three modalities. Then three feature matrices were organized as: fALFF (subjects by voxels: 905 × 78,420), GM (subjects by voxels: 905 × 76,969) and SNPs (905 × 4522), which corresponded to X_1_, X_2_, X_3_ in Fig. 1, respectively. These three matrices were normalized to ensure that all modalities had the same average sum-of-squares within each site and served as input to the subsequent multivariate fusion analysis. Site was coded as dummy variable and further regressed out together with age and gender for these modalities before multimodal fusion analysis.

### 2.5 Three-way para-ICA

The formed fALFF, GM and SNP matrices were jointly analyzed using three-way parallel independent component analysis (para-ICA) method, which estimates maximally independent component (IC) using ICA within each modality separately and maximizes the pair-wise correlations between modalities using an entropy term based on information theory [29]. The component extraction was based upon the Infomax algorithm [42]. Infomax extracts ICs through maximization of entropy which measures uncertainty associated with a random variable. As illustrated in Eq. (1), the cohort 1 were first decomposed into a linear combination of underlying components. *X*_1_, *X*_2_, *X*_3_ are observation matrices composed of measurements of fALFF, GM and SNP, that are the three *N*_subject_ × *N*_voxel/SNP_ matrices prepared above; *S*_1_, *S*_2_, *S*_3_ represent the unknown sources for each phenotype with each row representing an independent component; *A*_1_, *A*_2_, *A*_3_ are linear mixing matrices/loadings with each column representing a loading parameter associated with a specific component in *S*_1_, *S*_2_, *S*_3_; *W*_1_, *W*_2_, *W*_3_ are unmixing matrices. Based on the decomposition, the Infomax algorithm attempts to find the *W* matrix resulting in independence through maximizing an entropy function as defined in Eq. (2), where *f*_*y*_(*Y*) is the probability density function of *Y*; *E* is the expected value; *H* is the entropy function; *W*_0_ is the bias vector.

(1) $$\begin{array}{l} X_{1}=A_{1}\cdot S_{1}\overset{W_{1}=A_{1}^{-1}}{\to }S_{1}=W_{1}\cdot X_{1}\\ X_{2}=A_{2}\cdot S_{2}\overset{W_{2}=A_{2}^{-1}}{\to }S_{2}=W_{2}\cdot X_{2}\\ X_{3}=A_{3}\cdot S_{3}\overset{W_{3}=A_{3}^{-1}}{\to }S_{3}=W_{3}\cdot X_{3} \end{array}$$

(2) $$\begin{array}{l} \max\left\{H\left(Y\right)\right\}=-E\left\{\ln f_{y}\left(Y\right)\right\}\\ Y=\frac{1}{1+e^{-U}};U=\mathrm{WX}+W_{0} \end{array}$$

To enhance the inter-modality association, three-way para-ICA attempts to maximize the correlation calculated between the columns of the loadings matrices *A*_1_, *A*_2_, *A*_3_.Thus, three entropy terms and an additional correlation term comprise the objective function of three-way para-ICA, as shown in Eq. (3), where *f*_*i*, *j*, *k*_ is the additional aggregation function measuring the combined connection strength of the columns *i*, *j*, *k* from the corresponding matrices *A*_1_, *A*_2_, *A*_3_, respectively. This objective function will then be optimized through the gradient descent method. For a full description of the methodology and mathematical details of the algorithm, we refer readers to the original publications [29,43].

(3) $$\underset{\underset{i,j,k}{A_{1},A_{2},A_{3}}}{\arg\max}\left\{H\left(Y_{1}\right)+H\left(Y_{2}\right)+H\left(Y_{3}\right)+f_{i,j,k}\right\}$$

The code is available for public use through the Fusion ICA Toolbox (FIT, http://mialab.mrn.org/software/fit). Since the number of true sources from in-vivo data is in principal unknown, it is necessary to estimate the number of components before proceeding with the ICA factorization [29]. The number of components for the fALFF and GM data were estimated to be 13 and 10 respectively using a modified minimum description length criterion [44], a common method used in prior ICA based studies [45]. For the SNP data, an analogous method has been used [46], which ran ICA deposition with a large range of component numbers and drove a peak indicating the most reliable results to finally estimate the number to be 25.

### 2.6 Correlation and statistical analysis

Pair-wise correlations for all component pairs revealed by three-way para-ICA were evaluated, and significance levels were adjusted using Bonferroni correction for multiple comparisons. The most strongly correlated SNP-fALFF-GM link which passed Bonferroni correction was tested for group differences between HCs and SZs using two sample *t*-test, and the corresponding spatial components were further projected to cohort 2 to verify the inter-modality associations. The identified significant imaging and SNP components were converted to Z scores to select top contributing brain regions or SNPs for subsequent analyses.

### 2.7 Validation analysis

The spatial maps of the most significantly linked SNP-fALFF-GM pattern were projected to cohort 2, yielding the projected new loadings which were used to verify the pair-wise inter-modality association as [47] (Fig. 1). Specifically, we assumed that the spatial maps of the cohort 1 would generalize to the cohort 2. Thus by linear projection, we obtained the projected new loadings, as shown in Fig. S2. This process generated a set of subject-specific weights (*AA*_1_, *AA*_2_, *AA*_3_) for cohort 2 (*Y*_1_, *Y*_2_, *Y*_3_) that corresponded to the extent that a given subject's data from cohort 2 could be represented by the components determined from cohort 1. This was accomplished by multiplying (*Y*_1_, *Y*_2_, *Y*_3_) with the pseudoinverse of the source of cohort 1 (*S*_1_^−1^, *S*_2_^−1^, *S*_3_^−1^). The generated weights (*AA*_1_, *AA*_2_, *AA*_3_) for cohort 2 were then evaluated for inter-modality correlations to assess whether the pair-wise correlations between the identified ICs could be replicated in another independent cohort.

In the current study, stable antipsychotic treatment on the patients was not required at the moment of the MRI, so not all patients were on stable antipsychotic treatment at the moment of the MRI. But the PANSS total score was similar across two cohorts (*p* = .05). We then selected a subset of subjects in cohort 2, which consists of 49 SZ patients together with 87 HCs. The 49 patients did not show significant difference of PANSS scores compare with discovery data (p_postivie = 0.89, p_negative = 0.09). Then we ran same validating procedure by projecting spatial maps of discovery cohort 1 to the subset of cohort 2.

### 2.8 Genetic pathway analysis

Top contributing SNPs (|*Z*-scores| > 2) from the identified SNP component were annotated to the genes which were further applied Gene Ontology analysis by an online database WebGestalt (http://www.webgestalt.org/option.php). A hypergenometric test was used to detect an overrepresentation of the high-ranking genes among all the genes in a category. The whole genome was selected as the reference gene list. The significance values were FDR-adjusted to correct for multiple comparison.

### 2.9 Association with symptoms and cognitive performance

In addition, to enrich possible interpretation of the ICs identified by three-way para-ICA, we also assessed linear associations between working memory performance (DF and DB scores) /PANSS scores and para-ICA-derived components. Partial Pearson correlation was applied to compute the correlations between the identified ICs with working memory scores or PANSS scores separately, in which age, gender, site and diagnosis were controlled as covariates. Medication was regressed out on the 243 patients before computing the association.

### 2.10 Mediation analysis

Once a SNP-fALFF-GM pattern was identified to be group discriminative and associated with working memory performance (e.g. DB scores), as well as exhibiting significant pair-wise inter-modality correlation, we further conducted mediation analysis to better delineate the working pathways among them as shown in Fig. 1. Note that medication was regressed out on the imaging loading parameters of 243 patients before computing any mediation test. First, as previous studies have hinted us genetic factors may impact brain function through affecting structure [3, 27, 28, 48], we examined whether a mediation effect of SNP$\overset{\to \mathrm{GM}\to }{\to }$fALFF occurred. In this model, age, gender, site and diagnosis were considered as covariates. Then, as genetic effects on cognitive abilities were reported to be mediated by imaging phenotypes [26], we examined whether fALFF mediated SNP and DB scores (SNP$\overset{\to \text{fALFF}\to }{\to }$DB), as well as mediation of SNP and DB scores by GM (SNP$\overset{\to \mathrm{GM}\to }{\to }$DB). For the SNP$\overset{\to \text{fALFF}\to }{\to }$DB model, we set age, gender, site, diagnosis and GM as covariates. And for the SNP$\overset{\to \mathrm{GM}\to }{\to }$DB model, we regarded age, gender, site, diagnosis and fALFF as covariates. Furthermore, since brain structure has been previously reported to predict function, we tested whether GM could affect working memory performance through the mediation by fALFF (GM$\overset{\to \text{fALFF}\to }{\to }$DB) or directly. In this modal, age, gender, site and diagnosis were regarded as covariates.

In order to test the direct and indirect effects among the identified SNP, fALFF, GM and working memory scores, bootstrapping is one of the most valid and powerful methods [[49], [50], [51]]. It is a nonparametric resampling procedure, which does not impose the assumption of normality of the sampling distribution. Therefore, it can be applied to small samples with more confidence. Bootstrapping involves repeatedly sampling from the dataset and estimating the indirect effect (effects of the predictor on the outcomes through the mediators) in each resampled data set. PROCESS Macro [52] in SPSS v. 20 was used to conduct these analyses. This process was repeated 5000 times and provided estimates with a confidence interval (CI) of sampling distributions of the indirect effects, which maximized the statistical power. If the 95% CI does not contain zero, we conclude that the indirect effect is significantly different from zero at p < 0.05 and that mediation occurs.

## 3 Results

### 3.1 Linked SNP-fALFF-GM component

**Fig. 2:**
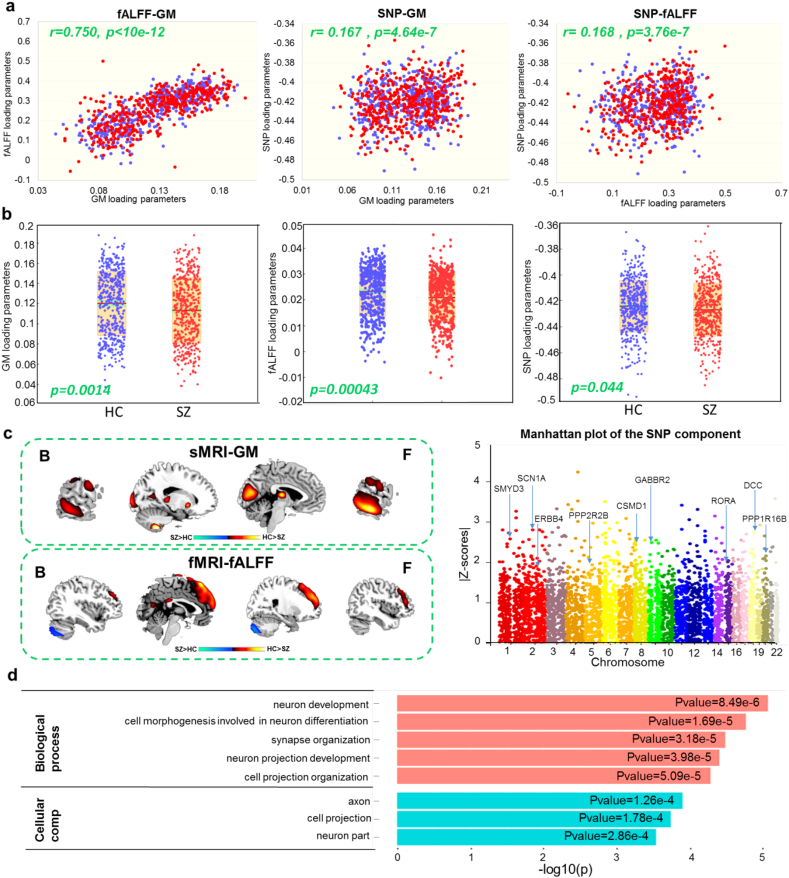
The strongest connected SNP-fALFF-GM component identified by three-way para-ICA. A: scatterplots of pair-wise correlation among the linked SNP-fALFF-GM pattern of SZ (red dot) and HC (blue dot) in cohort 1 after regressing diagnosis. B: group difference between SZ (red dot) and HC (blue dot) revealed by two sample *t*-test in each modality of cohort 1. C: the spatial maps visualized at |Z| > 2 for the imaging components and Manhattan plot for the SNP component. D: significant Gene Ontology analysis results revealed from 75 high-ranking genes, highlighting pathways like neuron development, synapse organization and axon pathways.

One strongest pair-wise correlated SNP-fALFF-GM triplet was identified in cohort 1. The pair-wise correlations between the linked components were fALFF-GM: *r* = 0.753, *p* < 10^−12^; SNP-fALFF: *r* = 0.174, *p* = 1.32 × 10^−7^; SNP-GM: *r* = 0.173, *p* = 1.78 × 10^−7^, passing Bonferroni-correction for multiple comparison. The correlations were consistent in 10-fold stability analysis with the average correlations of 0.713 ± 0.043, 0.179 ± 0.028, 0.180 ± 0.013 for fALFF-GM, SNP-fALFF and SNP-GM respectively. The identified linked pattern was robust to component numbers, as we observed similar components and inter-modality correlations when component number was set ±3 around the original number for each modality. After controlling for the diagnosis, partial correlations among the linked components were still significant (fALFF-GM: *r* = 0.750, *p* < 10^−12^; SNP-fALFF: *r* = 0.168, *p* = 3.76 × 10^−7^; SNP-GM: *r* = 0.167, *p* = 4.64 × 10^−7^, survived Bonferroni-correction, Fig. 2A), indicating similar associations among the triple components in both HCs and SZs. In order to control for ancestry effects, we performed PCA on the raw SNP data and generated the pairwise scatter plots of top PCs (Fig. S3). Moreover, we tested the group difference of each PC component using analysis of variance (ANOVA) across the seven sites (PC1: *p* = 5.67 × 10^−83^, PC2: *p* = 0.0076, PC3: *p* = 0.29, PC4: *p* = 0.53, PC5: *p* = 0.54). We then included the first three PCs as additional covariates and assessed the correlation between imaging modalities and SNP modality. The correlations between the two imaging components and the SNP component were still significant: SNP-fALFF: *r *= 0.148, *p* = 8.62 × 10^−6^; SNP-GM: *r* = 0.130, *p* = 9.80 × 10^−5^ (survived Bonferroni-correction). The less minor/reference allele counts on SNPs with positive component scores induce reduction of fALFF and GM. Furthermore, we conducted three-way para-ICA analysis on SNPs yielded by two other thresholds (*p* < 0.005 and *p* < .005) and those imaging features separately. In both *p* < 0.005 and *p* < 0.005 cases, the three-way pair-wise correlations between modalities were remained, and the fALFF and GM components show highly consistent spatial patterns. For SNP component, 52.5% overlap were obtained compared with *p* < 0.01. Note that although the associations could not survive correction for multiple comparisons, the SNP-fALFF-SNP pattern was similar to that identified with *p* < 0.01, as shown in Fig. S4.

In addition, age and gender in general are not expected to be significantly associated with autosomal SNPs. Therefore, we ran another analysis by regressing out age, gender and site for imaging modalities and regressing only site for the SNPs. The results are presented in Fig. S5. It can be seen that the three-way para-ICA can still yield a linked SNP-fALFF-GM pattern that was highly consistent between cohort 1 and cohort 2.

Since no significant correlation was observed between the identified imaging components and the medication dose measured by the chlorpromazine equivalent (p = 0.67, 0.84 for fALFF, GM) based on the 243 (out of 450) medicated patients, we further conducted a sub-set three-way para-ICA analysis on the 243 medicated SZ patients and 277 demographically-matched healthy controls. In this subset, medication was treated as nuisance covariate in the analysis both for fMRI and sMRI. The results are shown in Fig. S6, similar SNP-fALFF-GM pattern was revealed as well with significant correlation between modalities and significant group difference in each modality.

### 3.2 Group difference

Post hoc two sample *t*-test between HCs and SZs revealed significant group difference in the identified GM (*p* = 0.0014), fALFF (*p* = 0.0004) and SNP (*p* = 0.044) components respectively as shown in Fig. 2B. The loading plots in Fig. 2B were adjusted as HC > SZ for all modalities on the mean of loading parameters, so that the positive *Z*-values (red regions) indicated higher contribution in HC than SZ and the negative Z-values (blue regions) indicated higher contribution in SZ than HC. The spatial map of the GM component thresholded at |Z-score| > 2 was shown in Fig. 2C, with HC > SZ in thalamus, putamen, temporal gyrus, cuneus and cerebellum. Similarly, by thresholding the fALFF component with |Z-score| > 2, results indicated SZ had lower fALFF mainly in prefrontal gyrus, and higher fALFF in cerebellum compared with controls as shown in Fig. 2C. Anatomical information of the identified imaging components in details were shown in Table S3. The results for different threshold were also provided in the supplementary file (Fig. S7).

### 3.3 Validation

We then projected the spatial components of cohort 1 to cohort 2 as shown in Fig. 1, the resulted loadings in cohort 2 yielded significant pair-wise correlations (fALFF-GM: *r *= 0.680, *p* < 10^−12^; SNP-fALFF: *r *= 0.220, *p* = 0.0045; SNP-GM: *r* = 0.237, *p* = 0.0021, passing Bonferroni correction). When controlling for the diagnosis effect, the resulted loadings in cohort 2 again yielded significant pair-wise correlations (fALFF-GM: *r *= 0.667, *p* < 10^−12^; SNP-fALFF: *r* = 0.228, *p* = 0.0032; SNP-GM: *r* = 0.241, *p* = 0.0018, passing Bonferroni correction, Fig. S8-A). Meanwhile, we only observed significant group difference between SZ and HC in brain imaging data, *p* = 8.34 × 10^−4^, 0.024, 0.98 for fALFF, GM and SNP respectively (Fig. S8-B). In addition, when controlling for the diagnosis and medication effects, the resulted loadings in the subset of cohort 2 again yielded significant pair-wise correlations (fALFF-GM: *r* = 0.76, *p* < 10^–^^12^; SNP-fALFF: *r* = 0.22, *p* = 0.010; SNP-GM: *r *= 0.21, *p* = 0.014, Fig. S9), suggesting that the identified linked fALFF-GM-SNP pattern was reliable under multiple conditions.

### 3.4 Pathway analysis

Fig. 2C presents a Manhattan plot of the SNP component, highlighting genes including *CSMD1*, *CNTNAP2*, *DCC*, *GABBR2* etc. 172 SNPs were selected as top SNPs with |Z| > 2. Table S4 provides a summary of the identified 172 SNPs, including their corresponding genes and *Z*-scores. After annotating these SNPs, 75 genes were analyzed via a WebGestalt pathway analysis. Primarily significant Gene Ontology analysis results were provided in Fig. 2D, which demonstrated a significant enrichment of neuron development (*p* = 8.49 × 10^−6^), synapse organization (*p* = 3.18 × 10^−5^) and axon (*p* = 1.26 × 10^−4^) in our study. The full results of Gene Ontology analysis were provided in Table S5. The pathway results for different thresholds were also provided in the supplementary file (Table S6).

### 3.5 Association with cognitive measures

**Fig. 3:**
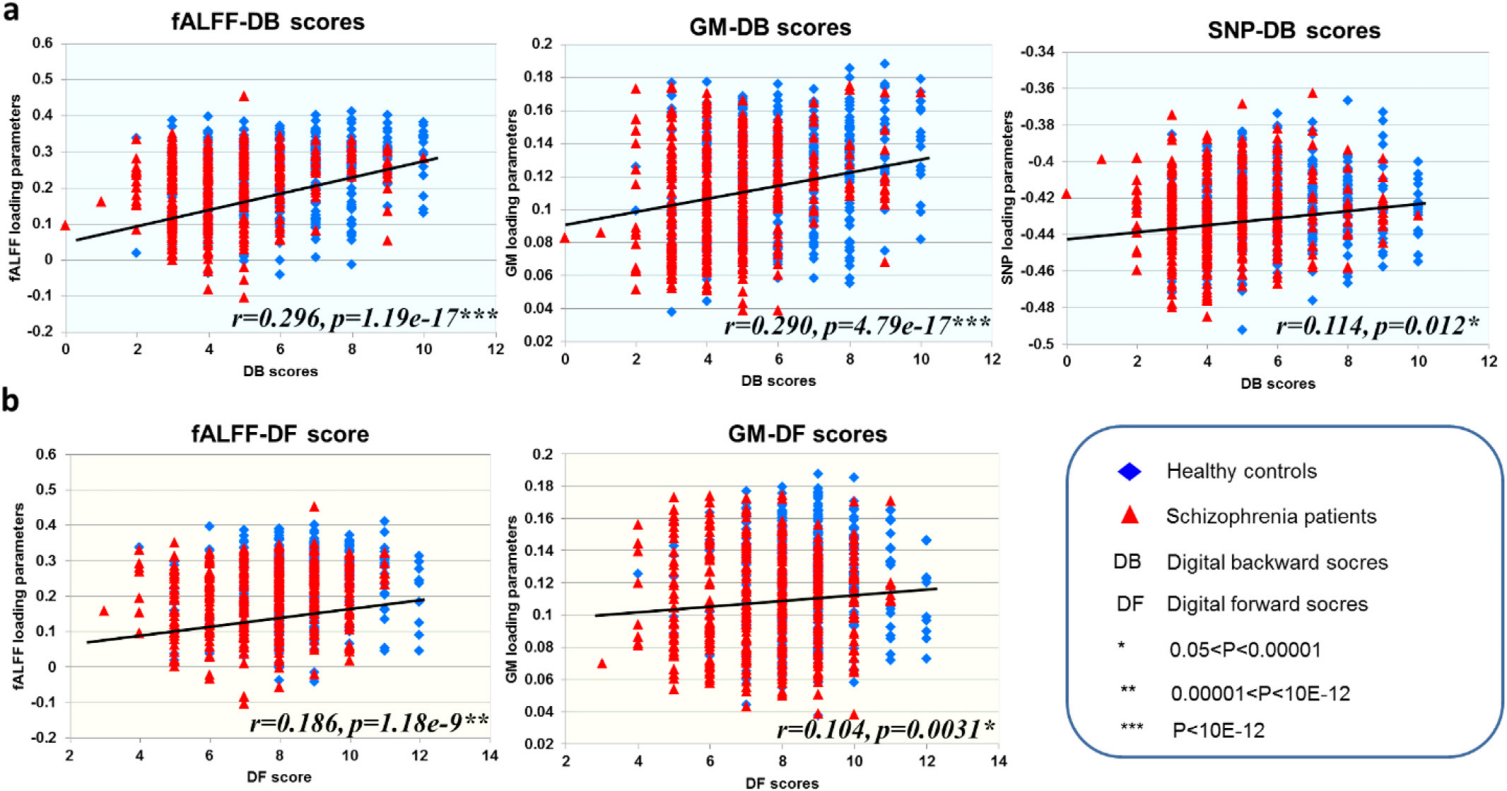
Correlation plots between loadings of the identified components and the scores. (A)(B) Correlation between loadings and digit backward [DB] scores (A) and digit forward [DF] scores (B); the higher loadings corresponded to better working memory performance. The results indicated that all the three modalities were significantly associated with DB scores. (C) Correlation between loadings and PANSS negative scores; the higher loadings corresponded to lower PANSS scores (less severe).

Fig. 3A and 3B present the positive correlations between the digital span performance and the joint components, in which higher DB and DF scores correspond to better working memory performance (fALFF-DB: *r* = 0.296, *p* = 1.19×10^−17^; GM-DB: *r* = 0.290, *p* = 4.79×10^−17^; SNP-DB: *r* = 0.114, *p* = 0.012; fALFF-DF: *r* = 0.186, *p* = 1.18×10^−9^; GM-DF: *r* = 0.104, *p* = 0.0031) after controlling for group difference. The association was still significant (fALFF-DB: *r* = 0.27, *p* = 2.89×10^−15^; GM-DB: *r *= 0.26, *p* = 7.69×10^−14^; fALFF-DF: *r* = 0.14, *p* = 3.44×10^−5^; GM-DF: *r* = 0.12, *p* = 5.32×10^−4^) when further controlling for medication effects on the GM and fALFF modalities. Results indicated higher GM volume in thalamus, putamen, temporal gyrus, cerebellum, higher fALFF in prefrontal gyrus, but lower fALFF in cerebellum and more minor/reference allele counts on SNPs were associated with better working memory performance. No significant correlation was found between PANSS and the identified SNP-fALFF-GM component.

### 3.6 Multiple mediation effects

Table S7-A indicated that both direct (CI = [0.02, 0.47]) and indirect effect (CI = [0.16, 0.60]) of SNP component on fALFF component via GM component was significant, which suggested a mediation effect presented as “$\mathrm{SNP}\overset{\to \mathrm{GM}\to }{\to }\text{fALFF}$” occured. Table S7-B indicated a significant indirect effect of SNP component on the DB scores via fALFF with confidence interval with CI from 0.05 to 1.93. However, the direct effect from SNP to DB after the addition of fALFF to the model was not significant, which suggested that SNP affected DB scores mainly through fALFF ($\mathrm{SNP}\overset{\to \text{fALFF}\to }{\to }\mathrm{DB}$) not the direct association. Since the 95% CI of the SNP$\overset{\to \mathrm{GM}\to }{\to }$DB model contained zero (CI = [−0.19, 1.13]) as shown in Table S7-C, the indirect effect was not significantly different from zero at the level of p < 0.05, therefore mediation effect was not remarkable. Table S7-D presented that GM can affect working memory directly or through mediation by fALFF with CI = [3.81, 10.82] ($\mathrm{GM}\overset{\to \text{fALFF}\to }{\to }\mathrm{DB}$). These results collectively indicated that the GM mediated the association between SNP and fALFF, while fALFF further mediated correlation between SNP, GM and working memory performance ($\mathrm{SNP}\overset{\to \mathrm{GM}\to }{\to }\text{fALFF}\to \text{cognition}$).

## 4 Discussion

**Fig. 4:**
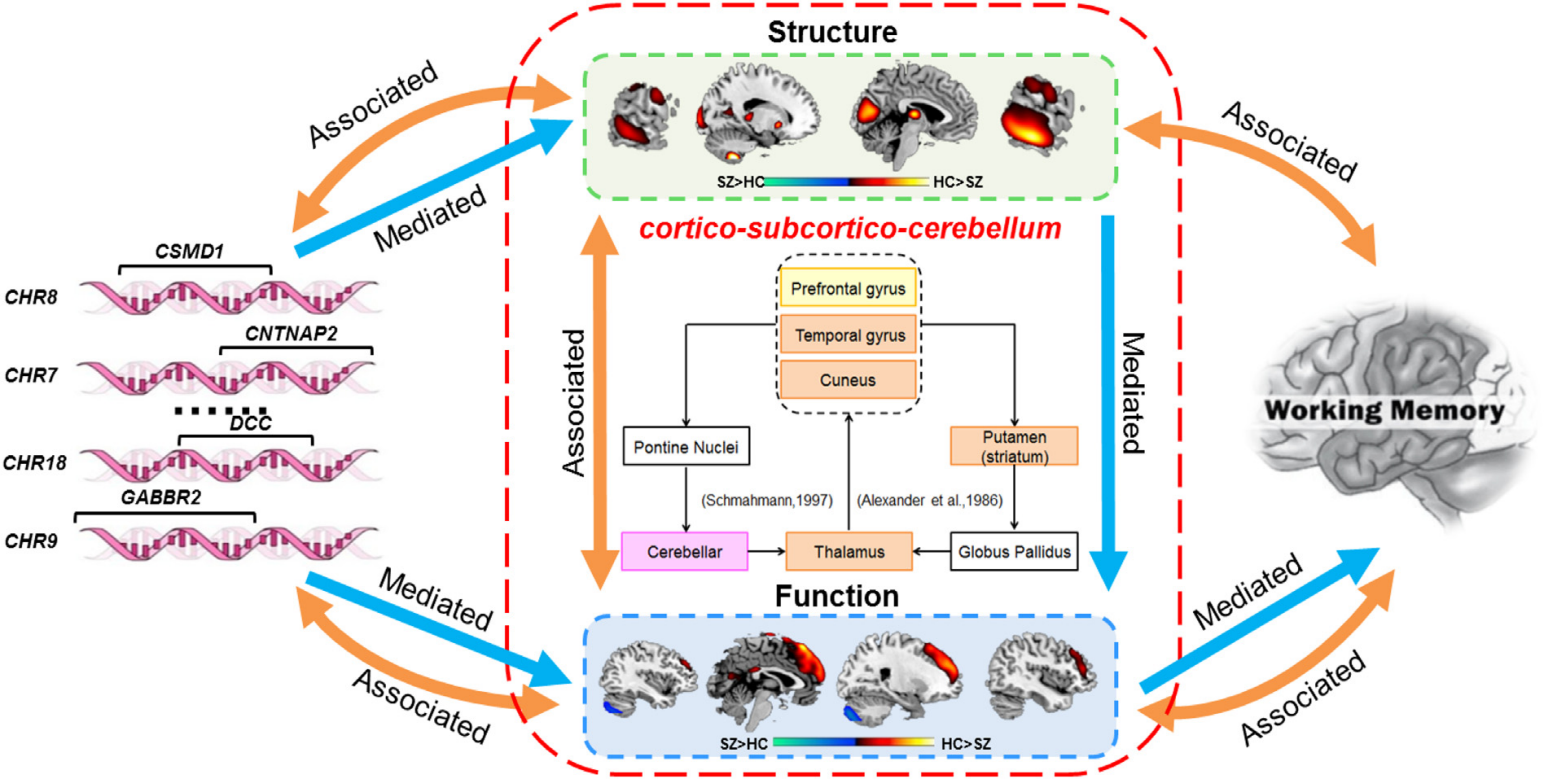
Summary figure of the identified schizophrenia-related Genetic-brain-cognition mediation pathway. GM reduction in thalamus, putamen, temporal gyrus, cuneus, cerebellum were correlated with fALFF alteration in prefrontal and cerebellum via cortico-subcortico-cerebellar neural circuit. Both imaging phenotypes were significantly correlated with genes like *CSMD1*, *CNTNAP2*, *DCC*, *GABBR2* etc. Further mediation tests suggested that the GM abnormalities significantly mediated the association between SNP and fALFF, while fALFF mediated the association between the genetic factors and the working memory performance.

To the best of our knowledge, this is the first study to jointly analyze fALFF, GM volume in conjunction with PGC-reported schizophrenia-susceptible SNPs in a large Chinese Han population using a multivariate, data-driven manner. One linked SNP-fALFF-GM component was identified to be both group discriminative and show significant correlation between modalities. Furthermore, post hoc mediation analyses suggested a significant “SNP$\overset{\to \mathrm{GM}\to }{\to }$fALFF→DB” relationship that was impaired in schizophrenia as Fig. 4.

On the lined components, GM volume reduction in schizophrenia was revealed in thalamus, putamen, bilateral temporal gyrus, cuneus and cerebellum, which has been supported by multiple studies [9,10,[53], [54], [55]]. Particularly, thalamus has been implicated as an important region for developing or maintaining appropriate synaptic connections with cortical targets and a critical hub for high-order cognition and cerebellum-cortical connection [56]. Putamen is an important part of dorsal striatum, which primarily mediates cognition involving motor function, certain executive functions and stimulus-response learning [57]. Additionally, the reduced volume in temporal gyrus is mainly responsible for multimodal sensory integration, high-order cognitive functions, higher-order aspects of language and voice information storage [[58], [59], [60]]. Abnormal GM volumes of cuneus are also widely reported in schizophrenia and have been suggested to underlie deficits in emotion perception and basic visual processing [61].

For the corresponding fALFF component, we observed reduced fALFF in prefrontal gyrus in SZ, which is consistent with other reported studies [20,62], as well as increased fALFF in cerebellum of SZs,in accordance with [63]. Interesting, the brain regions of the GM and fALFF modalities are part of the cortico-subcortico-cerebellum neural circuit which connecting the prefrontal cortex, the superior temporal sulcus, the basal ganglia and the cerebellum via the thalamus, which are believed to serve as neuroanatomical substrates of executive processing as described in [64].

On the genetic association, both imaging impairments in GM and fALFF are associated with genetic factors enriched in neuron development, synapse organization and axon pathways, highlighting genes including *CSMD1*, *CNTNAP2*, *DCC*, *GABBR2* etc. A recent study investigated the specific gene expression patterns arising from the 108 schizophrenia-associated loci also revealed that the genes in these loci were intensively enriched in the thalamus, cerebellum and cortex during several developmental stages [65]. The prefrontal activation during working memory tasks has been revealed to be both heritable and associated with calculated risk profile score from PGC's schizophrenia results [66]. *CSMD1* plays a significant role in the etiology of schizophrenia [67] and was reported to express in frontal-temporal and hippocampal regions of the brain by hierarchical bi-clustering analysis [68]. *CNTNAP2* was reported significantly associated with schizophrenia and major depression in the Han Chinese population [69] and highly expressed in frontal and anterior lobes, striatum and dorsal thalamus [70,71]. The reduced expression of *DCC* during development and/or throughout life confers resilience to the development of schizophrenia-like dopamine and behavioral abnormalities [72]. Expression of *GABA*_*B*_ receptor subunits 1 and 2 (*GABBR1* and *GABBR2*) were reported to alter significantly in the lateral cerebellum of subjects with schizophrenia [73]. The mRNA expression of *NFASC* is significantly downregulated in the superior temporal gyrus of persons with schizophrenia [74]. Biological function analysis based on the 75 high-ranking genes mainly indicated a significant enrichment of neuron development, synapse organization and axon. Schizophrenia is not an obstetric disease, but increasing evidence shows that impaired neural development is important in some cases [75]. Synapse and axon mainly participate in the process of information transmission [76]. In the context of a neurodevelopmental model, it is proposed that impaired mechanics of synaptic transmission in specific neural circuits during childhood and adolescence ultimately result in altered synapse formation or pruning, or both, which manifest in the clinical onset of schizophrenia [77,78].

More importantly, the identified associations were replicated in an independent Chinese Han dataset. In the validation, not only were the identified SNP-fALFF-GM spatial patterns similar between discovery and replication data, but the pair-wise association between 3 components were also replicated. In addition, we tried several strategies to control for age, medication differences across sites by running same analysis in several matched subsets. Appreciably, a similar SNP-fALFF-GM pattern could be found in all cases, suggesting that the identified imaging-genetic association impaired in SZ could be replicated in multiple conditions.

As to the association with cognition, when assessing the correlation between the identified SNP-fALFF-GM components and working memory performance (DF and DB scores), the imaging-genetic pattern were significantly associated with DB scores. Further mediation analyses revealed the GM mediated the association between SNP and fALFF, which was consistent with our hypothesis. Many studies have supported this finding by suggesting structure can determine or predict function [28,48] and structural features present a stronger association with genetic factors than function [3,27]. Moreover, fALFF significantly mediated correlation between SNP, GM and working memory performance. As SNP is functionally distant from cognition, the missing factors, e.g. methylation and mRNA, may cause to the un-significant direct effect between SNP and DB scores. The significant mediation effect between SNP and working memory performance via fALFF is supported by [[79], [80], [81]]. For example, an n-back working memory task fMRI study has detected that *SCN1A* allele-dependent activation differences in superior frontal gyrus typically involved in working memory processes [79]. Another high-ranking gene *CSMD1* was also proved to be associated with working memory through affecting prefrontal gyrus [80]. Furthermore, genetic variation in *PPP2R2B* was suggested to affect mRNA expression of this gene in prefrontal cortex to be associated with prefrontal processing during working memory [81]. Finally, since GM and fALFF regions were both included in the cortico-subcortico-cerebellum neural circuit, a possible speculation is that the brain regions with GM reduction may first affect fALFF decrease in prefrontal area via the cortico-subcortico-cerebellum neural circuit, and then caused to working memory impairment.

In addition, the current study conducted imaging-genetic analysis in a large Chinese Han population, which would raise a question that if it is reasonable to select risk SNPs based on the PGC GWAS. The explanations could be, first, the recent Chinese Han GWAS study [82] has claimed that the high-ranking risk loci in PGC's results appear to be largely consistent between the two populations. These observations lend support for our preselecting risk loci from PGC and applying them to the Chinese Han dataset. Notably, based on the preselected risk loci, we identified stable linked SNP-fALFF-GM components that showed schizophrenia relevance and replicated in an independent cohort, which indicates that these PGC selected risk loci do relate to schizophrenia as well as schizophrenia-discriminative structural and functional abnormalities. Thus, using PGC to conduct preselection of risk loci of schizophrenia does not appear to be a big concern. And future work can further verify the results only based on the Chinese GWAS results when the summary data is available.

Several limitations still need to be addressed in the present work. First, though no direct correlation was found between the incomplete medication information of antipsychotic dose and the identified components, medication may still have an underlying effect on multimodal imaging measures of schizophrenia patients as medication information for part of the patients were unavailable [63]. The second limitation is the power issue caused by the small size of cohort 2, which could result in the lack of a significant group difference in the SNP modality in cohort 2. Third, the cohort 2 were not perfectly matched with cohort 1 on medication, age and symptoms due to the site effect across a relatively large sample in cohort 1. However, subsequent validation tests based on multiple matched-subsets suggested that the identified linked fALFF-GM-SNP was reliable across multiple variables between cohorts. Finally, here the high-ranking genes were identified by annotation information. In the future work, we can conduct more analysis like eQTL to determine associated genes with the risk SNPs and then pathway analysis.

## 5 Conclusion

To the best of our knowledge, this is the first attempt to jointly analyze brain fALFF, GM in conjunction with PGC's reported SNPs in a large Chinese Han population (905 + 166 subjects), using a multivariate, data-driven approach. Results demonstrated GM reduction in thalamus, putamen, temporal gyrus, cuneus, cerebellum were correlated with fALFF alteration in prefrontal and cerebellum. Furthermore,both imaging phenotypes were significantly correlated with genetic factors enriched in neuron development, synapse organization and axon pathways. Moreover, subsequent mediation analysis supported our initial hypothesis of revealing a “$\mathrm{SNP}\overset{\to \mathrm{GM}\to }{\to }\text{fALFF}\to \text{cognition}$” pathway, implicating that the polygenic risk factors could exert impact on phenotypic measures from brain structure to function, thus might lead to cognitive impairment in schizophrenia.

## Funding

This work was supported by the Natural Science Foundation of China (No. 81471367, 61773380, 31771076, 91432302, 91732305 & 31620103905); the Strategic Priority Research Program of the Chinese Academy of Sciences (No. XDBS01040100); the Science Frontier Program of the Chinese Academy of Sciences (No. QYZDJ-SSW-SMC019); National Key R&D Program of China (No. 2017YFA0105203); Primary Research & Development Plan of Jiangsu Province (No. BE2016630); the National Institute of Health (No. R56MH117107, R01EB005846, R01MH094524, P20GM103472) and the National Science Foundation (No. 1539067).

The funder of the study played no role in study design, data collection, data analysis, data interpretation, or writing of the report. The corresponding authors had full access to all the data in current study and had final responsibility for the decision to submit for publication.

## Declarations of interests

The authors report no biomedical financial interests or potential conflicts of interest.

## Author contributions

NL, JS, JC, DL, TJ participated in the planning and design of the study. NL conducted the data analysis. NL, JS, JC wrote the paper. NL, KX, SL, JL preprocessed the data. JS, JC, DL, VDC, JL, VMV, YC, FZ, ZY, NZ, LF participate the results interpretation. MS, DZ and TJ design the protocol of data scanning and collection. FZ, LT, YX, SL contributed to the recruitment of the subjects in the Wuxi Mental Center. LL, YY, HZ contributed to the recruitment of the subjects in the Xinxiang Site. PL, LL, HY, JY, DZ contributed to the recruitment of subjects in the Beijing site. JC, HW contributed to the recruitment of the subjects in the Wuhan Site. YC, HW contributed to the recruitment of subjects in the Xijing Site. HG, PW contributed to the recruitment of subjects in the Zhumadian Site.

## Appendix A Supplementary data

Supplementary material Image 1
