# Supplementary material for: A Schizophrenia-Related Genetic-Brain-Cognition Pathway Revealed in a Large Chinese Population
Source: eBioMedicine. 2018 Oct 16;37:471–82. doi: 10.1016/j.ebiom.2018.10.009 (PMC6284414; doi:10.1016/j.ebiom.2018.10.009)
Supplement: Supplementary file 1 — Supplementary material [file mmc1.docx]

#### Supplementary File

##### **Table S1. Scanning parameters for all dataset**

|  | | **Cohort 1** | | | | | **Cohort 2** |
| --- | --- | --- | --- | --- | --- | --- | --- |
| **Type of 3T MRI scanner** | Center | PKUH6、HLG、XJ | HMS | HMG | ZMD | RWU | WX |
|  | Vendor | Siemens | Siemens | General Electric | General Electric | General Electric | Siemens |
|  | Model | Trio 3T | Verio 3T | Signa HDxt 3T | Signa HDxt 3T | Signa HDxt 3T | Trio 3T |
| **Resting state fMRI parameters** | Sequence | Echo planar imaging | Echo planar imaging | Echo planar imaging | Echo planar imaging | Echo planar imaging | Echo planar imaging |
|  | TR(ms) | 2000 | 2000 | 2000 | 2000 | 2000 | 2000 |
|  | TE(ms) | 30 | 30 | 30 | 30 | 30 | 30 |
|  | FA | 90° | 90° | 90° | 90° | 90° | 90° |
|  | FOV  (ms) | 220 ×220 | 220 ×220 | 220 ×220 | 220 ×220 | 220 ×220 | 220 ×220 |
|  | Matrix size | 64 × 64 | 64 × 64 | 64 × 64 | 64 × 64 | 64 × 64 | 64 × 64 |
|  | Slice thickness | 4 | 4 | 4 | 4 | 4 | 4 |
|  | Slice gap | 0.6 | 0.6 | 0.6 | 0.6 | 0.6 | 0.6 |
|  | Slice number | 33 | 33 | 33 | 33 | 33 | 33 |
|  | Voxel size  (mm^3^) | 3.44 × 3.44 × 4.60 | 3.44 × 3.44 × 4.60 | 3.44 × 3.44 × 4.60 | 3.44 × 3.44 × 4.60 | 3.44 × 3.44 × 4.60 | 3.44 × 3.44 × 4.60 |
|  | Volume | 240 | 240 | 240 | 180 | 240 | 240 |
|  | Orientation | Axial | Axial | Axial | Axial | Axial | Axial |
| **T1-weighted scan parameters** | Sequence | MPRAGE | MPRAGE | BRAVO | BRAVO | BRAVO | MPRAGE |
|  | TR(ms) | 2530 | 2530 | 8.06 | 6.78 | 7.79 | 2530 |
|  | TE(ms) | 3.44 | 2.43 | 3.12 | 2.49 | 3.0 | 3.44 |
|  | TI(ms) | 1100 | 1100 | 1100 | 1100 | 1100 | 1100 |
|  | FA | 7° | 7° | 7° | 7° | 7° | 7° |
|  | FOV  (mm^2^) | 256× 256 | 256× 256 | 256× 256 | 256× 256 | 256× 256 | 256× 256 |
|  | Matrix size | 256× 256×192 | 256× 256×192 | 256× 256×188 | 256× 256×188 | 256× 256×188 | 256× 256×192 |
|  | Slice thickness | 1 | 1 | 1 | 1 | 1 | 1 |
|  | Voxel size(mm^3^) | 1 | 1 | 1 | 1 | 1 | 1 |
|  | Orientation | Sagittal | Sagittal | Sagittal | Sagittal | Sagittal | Sagittal |

**Note:** TR: Repetition time; TE: Echo time; FA: flip angle; FOV: Field of view; TI: Inversion time; PKUH6:Peking University Sixth Hospital; HLG: Huilongguan Hospital; XJ: Xijing Hospital; HMS: Henan Mental Hospital Siemens scanning site; HMG: Henan Mental Hospital GE scanning site.; ZMD: Zhumadian Psychiatric Hospital; RWU: Remin Hospital of Wuhan University; WX: Wuxi Mental Health Center.

##### **Table S2. Demographic and clinical details of all the subjects for each site in the discovery cohort**

| Mean  (± SD ) | PANSS_p  (mean±sd) | PANSS_n  (mean±sd) | PANSS_g  (mean±sd) | PANSS_t  (mean±sd) | Digit span forward | | Digit span backward | |
| --- | --- | --- | --- | --- | --- | --- | --- | --- |
|  |  |  |  |  | HC | SZ | HC | SZ |
| PKUH6 | 23.75±4.44 | 18.42±5.78 | 35.51±5.24 | 77.68±9.07 | 8.76±1.49 | 8.72 ±1.31 | 5.95±1.86 | 5.44±1.73 |
| HLG | 25.98±2.82 | 16.33±3.13 | 35.67±3.35 | 77.98±5.95 | 9.00±1.32 | 8.25±1.30 | 7.16±1.62 | 5.13±1.70 |
| HMS | 24.41±3.56 | 22.94±6.37 | 39.76±5.74 | 87.11±11.29 | 8.18±1.42 | 6.52±1.62 | 5.49±1.62 | 3.92±1.21 |
| HMG | 22.64±2.60 | 19.88±5.64 | 40.12±4.94 | 82.64±8.89 | 8.28±1.06 | 6.44±1.12 | 5.97±1.58 | 4.52±1.15 |
| XJ | 23.21±4.82 | 22.73±7.71 | 45±9.74 | 90.94±17.31 | 9.29±0.96 | 8.67±1.18 | 8.79±1.11 | 6.42±2.40 |
| RWU | 23.88±4.43 | 20.85±5.12 | 43.47±7.41 | 87.78±12.03 | 9.14±1.11 | 7.91±1.21 | 6.47±1.46 | 3.93±1.19 |
| ZMD | 24.62±4.63 | 20.75±5.69 | 39.80±6.84 | 84.20±15.88 | 8.30±1.23 | 7.32±1.39 | 5.13±1.43 | 4.14±1.23 |

**Note:** PKUH6: Peking University Sixth Hospital; HLG: Huilongguan Hospital; XJ: Xijing Hospital; HMS: Henan Mental Hospital Siemens scanning site; HMG: Henan Mental Hospital GE scanning site.; ZMD: Zhumadian Psychiatric Hospital; RWU: Remin Hospital of Wuhan University.

##### **Table S3. Anatomical information of the identified imaging components at |Z|>2**

| sMRI-GM | | | |
| --- | --- | --- | --- |
| Area_HC>SZ | Brodmann Area | volume (cc) | random effects: Max Value (x, y, z) |
| Thalamus |  | 3.6/3.3 | 5.6 (-12, -26, 10)/5.5 (9, -26, 10) |
| Putamen |  | 1.7/0.9 | 5.4 (-21, 11, -3)/2.6 (21, 9, -3) |
| Cuneus | 7, 17, 18, 19, 23, 30 | 12.6/8.8 | 5.1 (0, -78, 18)/4.7 (3, -78, 15) |
| Precuneus | 23, 31 | 1.5/0.5 | 4.3 (0, -72, 23)/3.1 (3, -69, 23) |
| Middle Temporal Gyrus | 19, 20, 21, 22, 37, 39 | 6.4/12.4 | 3.2 (-65, -32, -3)/4.7 (62, -30, -11) |
| Inferior Temporal Gyrus | 18, 19, 20, 21, 37 | 0.3/2.6 | 2.1 (-62, -47, -8)/3.6 (62, -24, -16) |
| Superior Temporal Gyrus | 21, 22, 39, 42 | 1.2/3.5 | 2.6 (-65, -23, -1)/3.0 (59, -55, 11) |
| Precentral Gyrus | 4, 6, 44 | 4.3/6.3 | 2.8 (-59, -2, 28)/2.9 (59, -2, 22) |
| Postcentral Gyrus | 1, 2, 3, 40, 43 | 1.4/4.2 | 2.6 (-62, -10, 25)/2.9 (53, -18, 51) |
| Lingual Gyrus | 17, 18 | 2.4/1.0 | 3.3 (-3, -84, 4)/3.1 (3, -84, 4) |
| Inferior Parietal Lobule | 40 | 0.8/2.9 | 2.4 (-59, -30, 40)/3.0 (56, -47, 41) |
| Cerebellar |  | 3.5/3.6 | 5.6 (-15, -52, -40)/4.9 (15, -51, -38) |
| fMRI-fALFF | | | |
| Area_HC>SZ | Brodmann Area | volume (cc) | random effects: Max Value (x, y, z) |
| Superior Frontal Gyrus | 6, 8, 9, 10 | 15.4/21.8 | 2.8 (-21, 56, 22)/3.1 (27, 45, 25) |
| Middle/Medial/Inferior Frontal Gyrus | 6, 8, 9, 10, 46 | 8.6/21.8 | 2.7 (-24, 56, 19)/3.0 (30, 39, 28) |
| Cingulate Gyrus | 23, 31, 32 | 1.9/1.0 | 2.6 (0, -31, 26)/2.4 (6, 36, 29) |
| Posterior Cingulate/Precuneus | 23, 29, 31 | 0.8/0.3 | 2.4 (-3, -37, 24)/2.1 (3, -63, 17) |
| Area_HC<SZ |  |  |  |
| Cerebellar |  | 18.7/14.4 | 3.8 (-30, -80, -34)/3.7 (21, -83, -31) |

##### **Table S4. Summary of the identified 172 SNPs with |Z|>2**

| CHR | SNP | BP | MA | Gene | Z | CHR | SNP | BP | MA | Gene | Z |
| --- | --- | --- | --- | --- | --- | --- | --- | --- | --- | --- | --- |
| 1 | rs6695327 | 174061844 | A |  | 3.27 | 7 | rs1528523 | 147177691 | A | CNTNAP2 | 2.37 |
| 1 | rs10798282 | 173437024 | A | LOC100506023 | 3.10 | 7 | rs3815215 | 158704054 | A | WDR60 | 2.30 |
| 1 | rs7543044 | 35674311 | A |  | 2.80 | 7 | rs8043 | 128607384 | G | TNPO3 | 2.20 |
| 1 | rs5878 | 172145455 | A | DNM3 | 2.80 | 8 | rs1487745 | 18621332 | A | PSD3 | 2.56 |
| 1 | rs6677997 | 96954561 | A |  | 2.73 | 8 | rs11996192 | 109875397 | G |  | 2.55 |
| 1 | rs4839426 | 115784029 | A |  | 2.58 | 8 | rs2176903 | 13810119 | G |  | 2.52 |
| 1 | rs4654096 | 246391677 | G | SMYD3 | 2.50 | 8 | rs12541020 | 4817592 | G | CSMD1 | 2.47 |
| 1 | rs11161570 | 85721911 | A | C1orf52 | 2.47 | 8 | rs10503478 | 13832082 | A |  | 2.43 |
| 1 | rs11163210 | 81670050 | A |  | 2.38 | 8 | rs386310 | 16039922 | A | MSR1 | 2.31 |
| 1 | rs2595968 | 204977799 | G | NFASC | 2.36 | 8 | rs10958432 | 55761124 | A |  | 2.14 |
| 1 | rs1194596 | 154238383 | A | UBAP2L | 2.23 | 8 | rs17118702 | 13979289 | G | SGCZ | 2.06 |
| 1 | rs10754693 | 240314499 | G | FMN2 | 2.19 | 8 | rs13274039 | 8111659 | A |  | 2.04 |
| 1 | rs10920090 | 200972454 | G | KIF21B | 2.17 | 8 | rs1462436 | 76804524 | A |  | -2.03 |
| 1 | rs490285 | 182705001 | A |  | 2.15 | 9 | rs4287017 | 83309521 | A |  | 2.55 |
| 1 | rs11488569 | 38111382 | A |  | 2.02 | 9 | rs855508 | 33617310 | G |  | 2.49 |
| 1 | rs10752747 | 2524915 | C | MMEL1 | 2.02 | 9 | rs12554364 | 101235046 | G | GABBR2 | 2.48 |
| 2 | rs810057 | 97015073 | G | NCAPH | 2.81 | 9 | rs2184026 | 101304348 | G | GABBR2 | 2.22 |
| 2 | rs1542484 | 166905375 | A | SCN1A | 2.80 | 9 | rs10811561 | 21530609 | G | MIR31HG | 2.19 |
| 2 | rs4673905 | 201163783 | G |  | 2.76 | 9 | rs1885431 | 10225533 | G | PTPRD | 2.03 |
| 2 | rs6436755 | 228999691 | A | SPHKAP | 2.62 | 10 | rs239865 | 115685187 | A |  | 2.33 |
| 2 | rs17047515 | 56376801 | G |  | 2.57 | 10 | rs7898793 | 126284935 | A | LHPP | 2.31 |
| 2 | rs2028201 | 201303010 | A | SPATS2L | 2.41 | 10 | rs7921902 | 55841776 | G | PCDH15 | 2.28 |
| 2 | rs13020778 | 102784574 | G | IL1R1 | 2.40 | 10 | rs12775823 | 4647269 | A |  | 2.15 |
| 2 | rs13015993 | 217625523 | G |  | 2.31 | 11 | rs7122539 | 66662731 | A | PC | 3.42 |
| 2 | rs1484465 | 80298707 | G | CTNNA2 | 2.16 | 11 | rs896436 | 66781263 | G |  | 2.81 |
| 2 | rs6720427 | 2527641 | G |  | -2.14 | 11 | rs1620329 | 132577443 | A | OPCML | 2.23 |
| 2 | rs7592506 | 79420044 | A |  | 2.14 | 11 | rs1994373 | 24744592 | G | LUZP2 | 2.16 |
| 2 | rs4491670 | 147591712 | A |  | 2.11 | 11 | rs260815 | 103918740 | G | PDGFD | 2.08 |
| 2 | rs17479466 | 96962859 | G | SNRNP200 | -2.07 | 11 | rs3862800 | 74152044 | G |  | 2.01 |
| 2 | rs10201223 | 12272871 | C |  | 2.04 | 12 | rs9667991 | 95179862 | C |  | 3.32 |
| 2 | rs4233806 | 166996841 | A | SCN1A | 2.03 | 12 | rs2954932 | 12084073 | A |  | 3.06 |
| 3 | rs16851083 | 105072927 | A |  | 3.32 | 12 | rs10507045 | 95252088 | G |  | 2.90 |
| 3 | rs13076885 | 122617445 | A |  | 2.87 | 12 | rs2728804 | 17564072 | C |  | 2.46 |
| 3 | rs2123184 | 56271889 | A | ERC2 | 2.74 | 12 | rs7953274 | 87901028 | A |  | 2.42 |
| 3 | rs10936928 | 176785767 | C | TBL1XR1 | 2.67 | 12 | rs1000702 | 95256296 | C |  | 2.27 |
| 3 | rs4142769 | 159173074 | A | SCHIP1 | 2.64 | 12 | rs7294369 | 95194443 | A |  | 2.15 |
| 3 | rs280534 | 193425749 | A |  | 2.63 | 12 | rs2555277 | 119585950 | G | SRRM4 | 2.15 |
| 3 | rs6439680 | 136763653 | A |  | 2.43 | 12 | rs6489498 | 3902269 | G |  | 2.07 |
| 3 | rs12487571 | 25004369 | A |  | 2.41 | 12 | rs1798255 | 32287259 | G | BICD1 | 2.02 |
| 3 | rs749613 | 2552565 | G | CNTN4 | 2.23 | 12 | rs1144713 | 32261239 | G | BICD1 | 2.02 |
| 3 | rs775022 | 10404739 | C | ATP2B2 | 2.20 | 12 | rs17220075 | 13681321 | G |  | 2.01 |
| 3 | rs9828864 | 80809059 | A |  | 2.08 | 13 | rs7491050 | 44259766 | A | ENOX1 | 2.77 |
| 3 | rs2371150 | 27806147 | A |  | 2.06 | 13 | rs9532985 | 42644377 | A | DGKH | 2.65 |
| 3 | rs1458062 | 56016020 | G | ERC2 | 2.03 | 13 | rs895266 | 44461179 | A | LACC1 | 2.55 |
| 4 | rs11945758 | 118667234 | C |  | 4.25 | 13 | rs1466005 | 44424210 | G | CCDC122 | 2.50 |
| 4 | rs6534063 | 118803489 | A |  | 3.53 | 13 | rs1572592 | 102036107 | A | NALCN | 2.28 |
| 4 | rs4525972 | 19492256 | G |  | 3.43 | 13 | rs9594333 | 40057973 | A | LHFP | 2.04 |
| 4 | rs1606137 | 60268638 | A |  | 3.32 | 14 | rs1998192 | 29444668 | A |  | 3.15 |
| 4 | rs4597906 | 118758795 | G |  | 3.02 | 14 | rs8009019 | 103769595 | C |  | 2.86 |
| 4 | rs4579126 | 97737582 | A |  | 2.60 | 14 | rs10149831 | 42187916 | G | LRFN5 | 2.60 |
| 4 | rs10032784 | 190268759 | G |  | 2.53 | 14 | rs10147990 | 93703854 | A |  | 2.48 |
| 4 | rs17039386 | 161159633 | A |  | 2.37 | 14 | rs225882 | 30480123 | G |  | 2.26 |
| 4 | rs13129234 | 58474857 | A |  | 2.28 | 14 | rs12586589 | 55996622 | G |  | 2.09 |
| 4 | rs1288547 | 186216136 | G | SNX25 | 2.18 | 14 | rs2295148 | 104195610 | G | ZFYVE21 | 2.08 |
| 4 | rs4145 | 138118550 | G |  | 2.02 | 15 | rs2925235 | 25775992 | A |  | 2.28 |
| 5 | rs10059045 | 85687037 | A |  | 2.97 | 15 | rs573922 | 58740094 | G | LIPC | 2.05 |
| 5 | rs256209 | 132971131 | A |  | 2.63 | 15 | rs17651808 | 52872321 | A |  | 2.02 |
| 5 | rs10940072 | 65916784 | G | MAST4 | 2.37 | 16 | rs3096324 | 89422823 | A | ANKRD11 | 2.55 |
| 5 | rs10941370 | 37833419 | G | GDNF | 2.14 | 16 | rs9938207 | 6729357 | A | RBFOX1 | 2.31 |
| 6 | rs396660 | 29646165 | A |  | 3.50 | 16 | rs9939407 | 6796866 | G | RBFOX1 | 2.10 |
| 6 | rs6916187 | 31463926 | G |  | 2.99 | 16 | rs8055853 | 55078667 | G |  | 2.07 |
| 6 | rs789830 | 150408096 | A |  | 2.98 | 16 | rs8056716 | 13643974 | G |  | 2.03 |
| 6 | rs1328706 | 72453286 | A |  | 2.84 | 17 | rs244363 | 53188893 | A | STXBP4 | 2.70 |
| 6 | rs17843604 | 32620283 | G |  | 2.72 | 17 | rs8067705 | 20819995 | G |  | 2.52 |
| 6 | rs191833 | 5482597 | A | FARS2 | 2.68 | 17 | rs12603276 | 34946547 | G |  | 2.26 |
| 6 | rs9270911 | 32572202 | G |  | 2.60 | 17 | rs11870213 | 13977462 | A | COX10 | 2.24 |
| 6 | rs6900525 | 110239670 | G |  | 2.52 | 17 | rs12943477 | 13815860 | C |  | 2.09 |
| 6 | rs3130630 | 31510933 | C | DDX39B | 2.49 | 17 | rs17730540 | 6255977 | A |  | -2.04 |
| 6 | rs6915845 | 131422563 | G |  | 2.49 | 18 | rs8098283 | 50208623 | A | DCC | 2.73 |
| 6 | rs444697 | 33575009 | A |  | 2.46 | 18 | rs17223656 | 23100817 | A |  | 2.33 |
| 6 | rs11751562 | 149080301 | G | UST | 2.25 | 18 | rs12606744 | 36943919 | G | LINC00669 | 2.33 |
| 6 | rs9356265 | 165173588 | A |  | 2.20 | 18 | rs1451289 | 22390204 | C |  | 2.23 |
| 6 | rs640941 | 65092688 | G | EYS | 2.15 | 18 | rs9964078 | 52384590 | A |  | 2.20 |
| 6 | rs241438 | 32797620 | A | TAP2 | 2.15 | 18 | rs7238305 | 51374587 | A |  | 2.13 |
| 6 | rs2756184 | 43234316 | C | TTBK1 | 2.14 | 19 | rs7255838 | 31207592 | G |  | 2.93 |
| 6 | rs2071476 | 32825379 | G | PSMB9 | 2.13 | 20 | rs6047233 | 21059248 | C |  | 2.66 |
| 6 | rs3828867 | 16722436 | A | ATXN1 | 2.12 | 20 | rs809220 | 21414037 | G |  | -2.34 |
| 6 | rs2022265 | 84293271 | A | SNAP91 | 2.06 | 20 | rs220501 | 37297126 | C |  | 2.16 |
| 6 | rs3813355 | 132910612 | G | TAAR5 | 2.05 | 20 | rs1885284 | 21470454 | G |  | -2.10 |
| 6 | rs1935676 | 112695882 | A |  | 2.04 | 20 | rs6037443 | 2961408 | G | PTPRA | 2.09 |
| 6 | rs1744506 | 165625891 | G |  | 2.02 | 20 | rs208817 | 37492316 | G | PPP1R16B | 2.04 |
| 6 | rs9347879 | 165015261 | A |  | 2.02 | 21 | rs4624474 | 40686327 | A | BRWD1-IT2 | 2.38 |
| 7 | rs12665877 | 78344461 | A | MAGI2 | 3.09 | 21 | rs2835289 | 37535897 | A |  | 2.37 |
| 7 | rs6942710 | 16590306 | A | LRRC72 | 2.79 | 21 | rs2824065 | 18187408 | G |  | 2.26 |
| 7 | rs193795 | 105621512 | C | CDHR3 | 2.70 | 22 | rs982520 | 19310812 | A |  | 3.59 |
| 7 | rs214463 | 110854261 | C | IMMP2L | 2.58 | 22 | rs5762942 | 29390049 | A | ZNRF3 | 2.00 |

**Note**: Chr: Chromosome; MA: minor allele.

##### **Table S5. GO analysis results of high-ranking genes correspond to |Z|>2 scored SNPs**

| p-value | q-value | Term_name | | Gene Symbol |
| --- | --- | --- | --- | --- |
| Biological Process | | | | |
| 8.49e-06 | 7.23e-02 | neuron development | UST;LRFN5;CTNNA2;CNTN4;DCC;NFASC;CNTNAP2;DNM3;GDNF;ATP2B2;OPCML;PTPRA;PTPRD;MAGI2 | |
| 1.69e-05 | 7.23e-02 | cell morphogenesis involved in neuron differentiation | UST;LRFN5;CTNNA2;CNTN4;DCC;NFASC;DNM3;ATP2B2;PTPRA;PTPRD | |
| 3.18e-05 | 8.51e-02 | synapse organization | CTNNA2;NFASC;DNM3;GDNF;ATP2B2;PTPRD;MAGI2 | |
| 3.98e-05 | 8.51e-02 | neuron projection development | UST;LRFN5;CTNNA2;CNTN4;DCC;NFASC;CNTNAP2;DNM3;GDNF;PTPRA;PTPRD;MAGI2 | |
| 5.09e-05 | 8.7e-02 | cell projection organization | UST;LRFN5;CTNNA2;CNTN4;DCC;NFASC;CNTNAP2;PPP1R16B;DNM3;GDNF;ATP2B2;WDR60;PTPRA;PTPRD;MAGI2 | |
| Cellular Component | | | | |
| 1.26e-04 | 9.15e-02 | axon | CTNNA2;CNTN4;DCC;KIF21B;NFASC;CNTNAP2;DNM3 | |
| 1.78e-04 | 9.15e-02 | cell projection | CTNNA2;CNTN4;DCC;KIF21B;NFASC;CNTNAP2;PPP1R16B;DNM3;ERC2;ATP2B2;WDR60;FMN2;PCDH15;GABBR2;MAGI2 | |
| 2.86e-04 | 9.83e-02 | neuron part | CTNNA2;CNTN4;DCC;KIF21B;NFASC;CNTNAP2;DNM3;ERC2;ATP2B2;PCDH15;GABBR2;MAGI2 | |

##### **Table S6. GO analysis of high-ranking genes corresponding to |Z|>2.1, 2.2 and 2.3 scored SNPs**

| GO_Term | p-value | q-value | Term_name [be consistent in red below] |
| --- | --- | --- | --- |
| Biological Process (\|Z\|>2.1) | 1.84e-05 | 1.58e-01 | neuron development |
|  | 1.22e-04 | 2.05e-01 | cell morphogenesis involved in neuron differentiation |
|  | 8.18e-05 | 2.05e-01 | synapse organization |
|  | 1.26e-04 | 2.05e-01 | neuron projection development |
|  | 1.32e-04 | 2.05e-01 | protein localization to axon |
|  | 1.44e-04 | 2.05e-01 | Neuron recognition |
|  | 1.72e-04 | 2.1e-01 | Neuron differentiation |
|  | 2.49e-04 | 2.66e-01 | Cell development |
|  | 2.95e-04 | 2.8e-01 | cell projection organization |
| Biological Process (\|Z\|>2.2) | 4.71e-05 | 2.26e-01 | neuron development |
|  | 1.48e-04 | 3.17e-01 | cell morphogenesis involved in neuron differentiation |
|  | 4.77e-04 | 4.54e-01 | neuron projection development |
|  | 7.93e-05 | 2.26e-01 | protein localization to axon |
|  | 6.71e-05 | 2.26e-01 | Neuron recognition |
|  | 3.17e-04 | 4.22e-01 | Neuron differentiation |
|  | 5.32e-04 | 4.54e-01 | Transmission of nerve impulse |
|  | 5.03e-04 | 4.54e-01 | cell projection organization |
| Biological Process (\|Z\|>2.3) | 5.87e-03 | >1 | neuron development |
|  | 2.68e-03 | >1 | neuron projection development |
|  | 5.86e-3 | >1 | cell projection organization |
| Cellular Component (\|Z\|>2.1) | 2.6e-05 | 1.28e-02 | axon |
|  | 3.74e-05 | 1.28e-02 | cell projection |
|  | 2.31e-05 | 1.28e-02 | neuron part |
|  | 5.22e-05 | 1.34e-02 | Neuron projection |
|  | 3.34e-04 | 6.89e-02 | synapse |
|  | 9.38e-04 | 1.23e-01 | Postsynaptic density |
| Cellular Component (\|Z\|>2.2) | 7.88e-04 | 1.62e-01 | axon |
|  | 4.33e-04 | 1.44e-01 | cell projection |
|  | 1.06e-04 | 1.09e-01 | neuron part |
|  | 3.83e-04 | 1.44e-01 | Neuron projection |
|  | 5.59e-04 | 1.44e-01 | synapse |
| Cellular Component (\|Z\|>2.3) | 2.26e-03 | 3.31e-01 | axon |
|  | 2.06e-03 | 3.31e-01 | neuron part |
|  | 3.66e-04 | 3.31e-01 | Neuron projection |
|  | 2.08e-03 | 3.31e-01 | synapse |

Note: The red term_names are the replicated pathways in different thresholds of the SNP component.

##### **Table S7. The direct and indirect effects revealed by the mediation analysis.**

| **A-1 Direct effect of SNP on fALFF*** | | | | | | | | | |
| --- | --- | --- | --- | --- | --- | --- | --- | --- | --- |
| Effect | SE | | t | | P | | LLCI | | ULCI |
| 0.25 | 0.11 | | 2.17 | | **0.03** | | 0.02 | | 0.47 |
| **A-2 Indirect effect of SNP on fALFF via GM*** | | | | | | | | | |
|  | | Effect | | Boot SE | | BootLLCI | | BootULCI | |
| GM | | 0.39 | | 0.11 | | 0.16 | | 0.60 | |
| **B-1 Direct effect of SNP on DB** | | | | | | | | | |
| Effect | SE | | t | | P | | LLCI | | ULCI |
| 4.09 | 3.05 | | 1.34 | | 0.18 | | -1.90 | | 10.09 |
| **B-2 Indirect effect of SNP on DB via fALFF*** | | | | | | | | | |
|  | | Effect | | Boot SE | | BootLLCI | | BootULCI | |
| fALFF | | 0.80 | | 0.47 | | 0.05 | | 1.93 | |
| **C-1 Direct effect of SNP on DB** | | | | | | | | | |
| Effect | SE | | t | | P | | LLCI | | ULCI |
| 4.09 | 3.05 | | 1.34 | | 0.18 | | -1.90 | | 10.09 |
| **C-2 Indirect effect of SNP on DB via GM** | | | | | | | | | |
|  | | Effect | | Boot SE | | BootLLCI | | BootULCI | |
| GM | | 0.24 | | 0.30 | | -0.19 | | 1.13 | |
| **D-1 Direct effect of GM on DB*** | | | | | | | | | |
| Effect | SE | | t | | P | | LLCI | | ULCI |
| 6.58 | 2.92 | | 2.26 | | **0.02** | | 0.86 | | 12.31 |
| **D-2 Indirect effect of GM on DB via fALFF*** | | | | | | | | | |
|  | | Effect | | Boot SE | | BootLLCI | | BootULCI | |
| fALFF | | 7.27 | | 1.83 | | 3.81 | | 10.82 | |

**Note:** **(A)** Mediation analysis results of direct and indirect effects of the SNP component on the fALFF via GM component. **(B)** Mediation analysis results of direct and indirect effects of the SNP component on the DB scores via fALFF component. **(C)** Mediation analysis results of direct and indirect effects of the SNP component on the DB scores via GM component. **(D)** Mediation analysis results of direct and indirect effects of the GM component on the DB scores via fALFF component. The digital interval [LLCI, ULCI] and [BootLLCI, BootULCI] represent the confidence intervals. CI is short for confidence interval. The * represent the significant effect (i.e., zeros is not included in the confidence intervals [p<0.05]).

**Supplementary Figures**

**
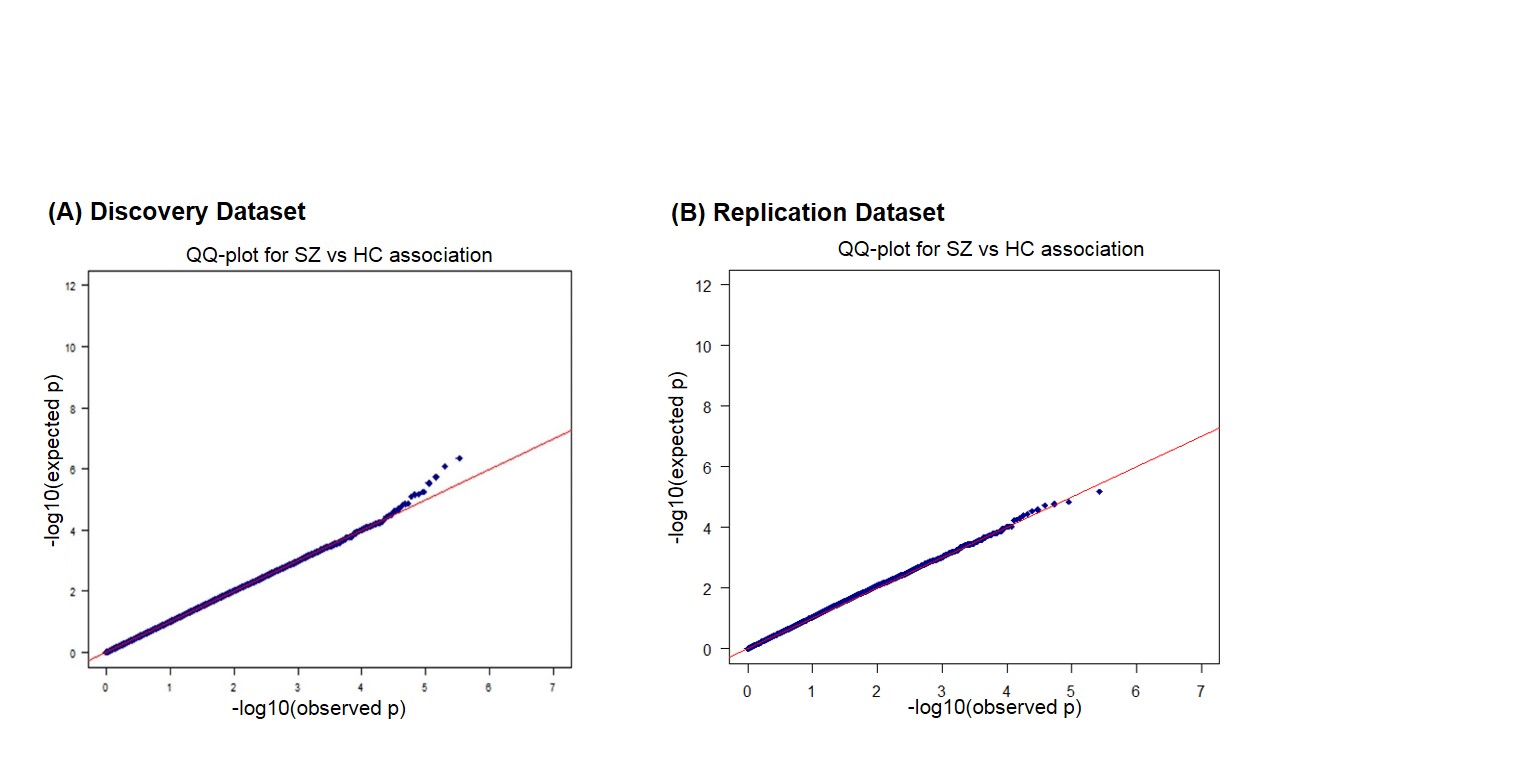
**

##### **Fig.S1 Quantile-Quantile plot.** Quantile-Quantile plot of p-values (ANOVA, group difference between patients and controls in terms of MAF) tested against a uniform distribution in the discovery dataset (A) and the replication dataset (B).


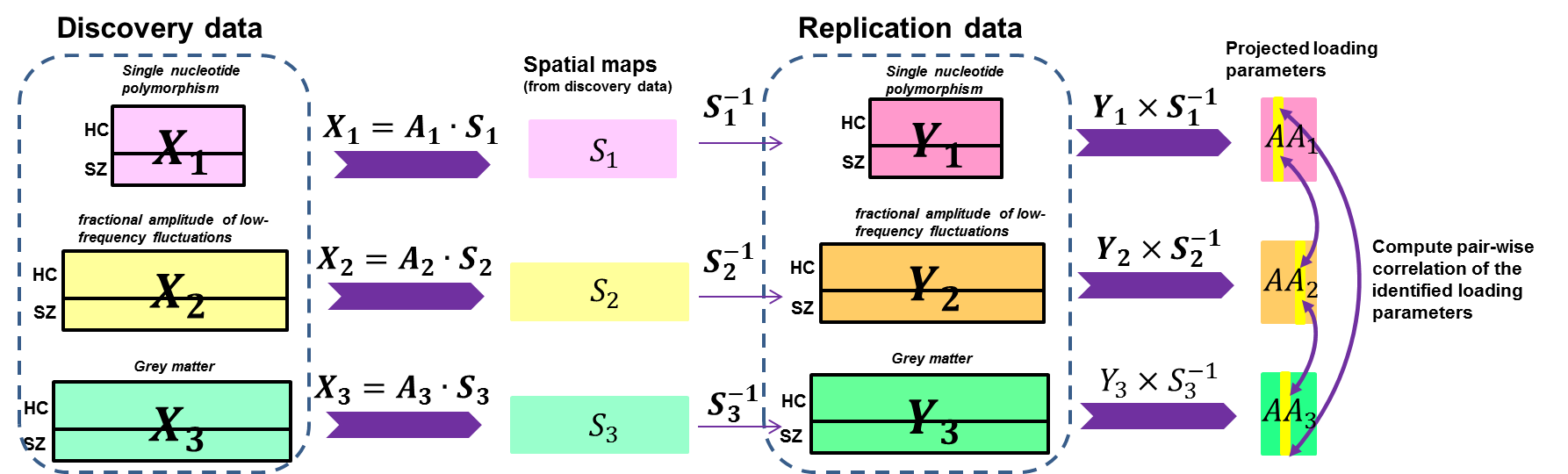


##### **Fig.S2 Pipeline of the replication the results without the use of ICA.**


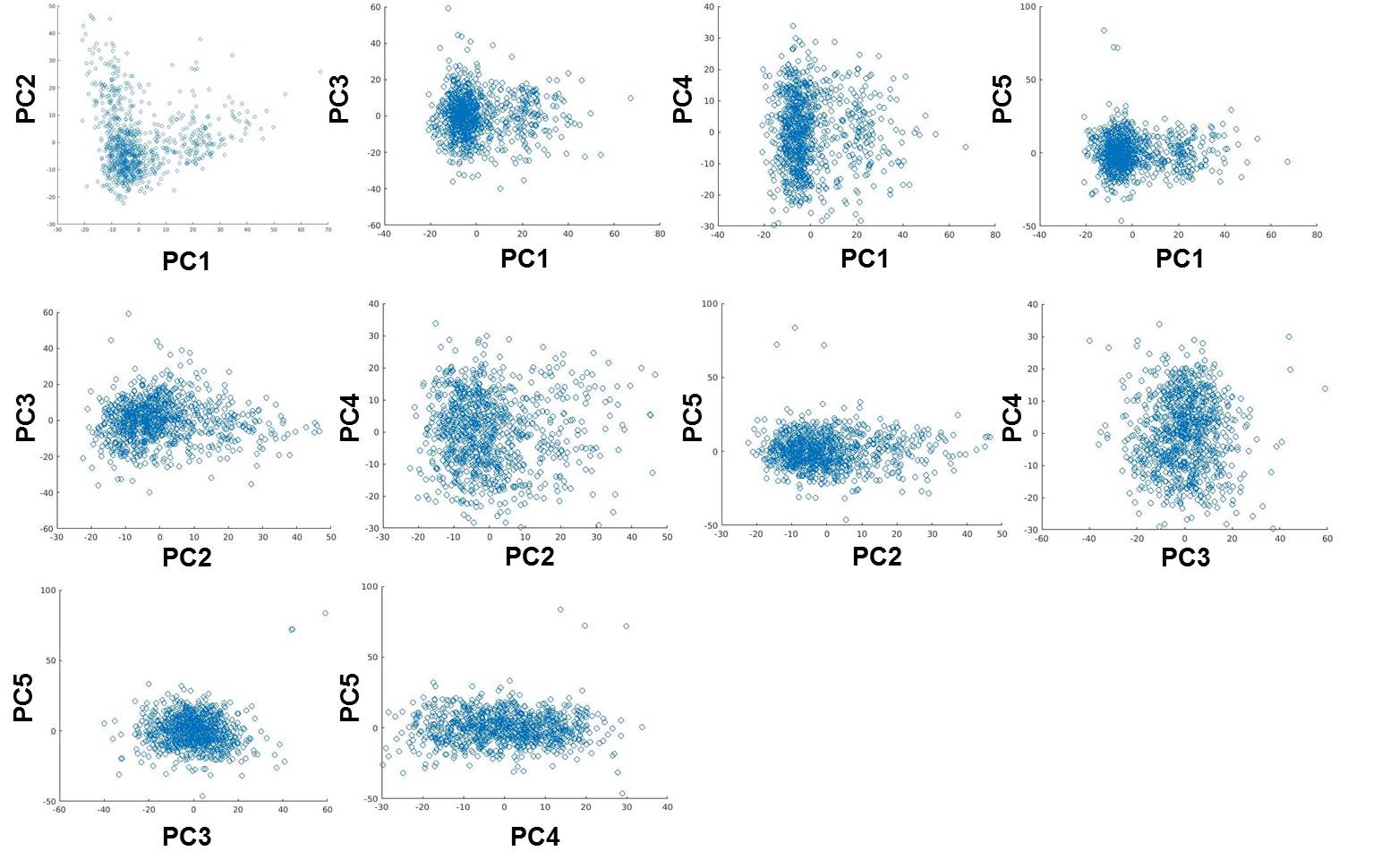


##### **Fig.S3 Correlations of the first five principle components**


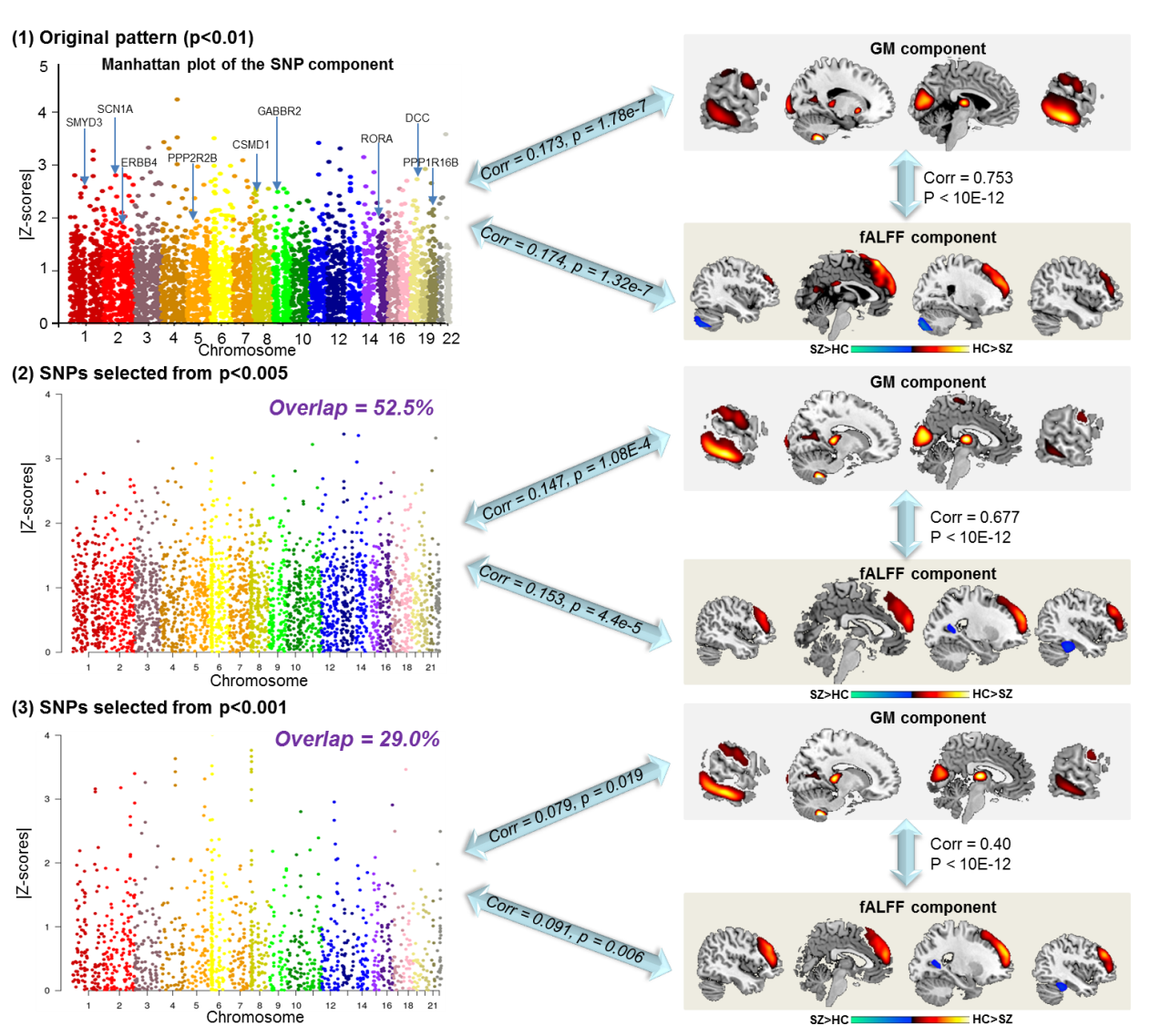


**Fig.S4 Three-way para-ICA based on different threshold of p-value**


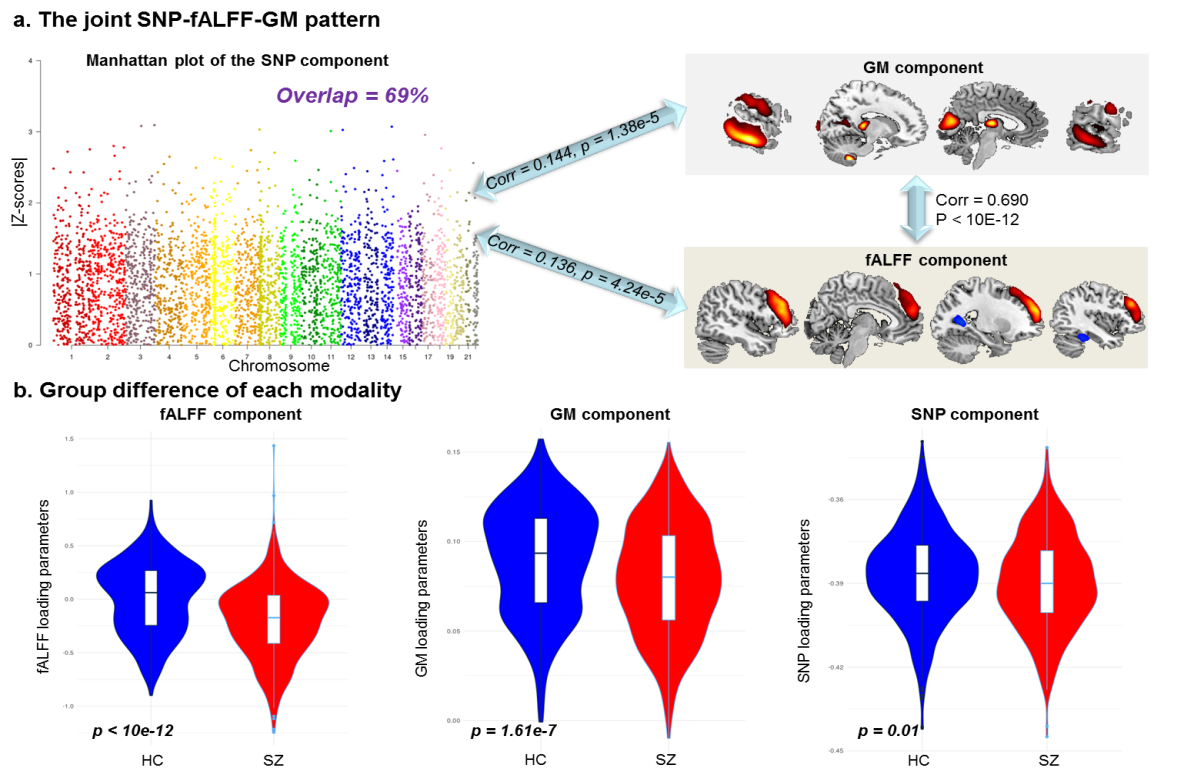


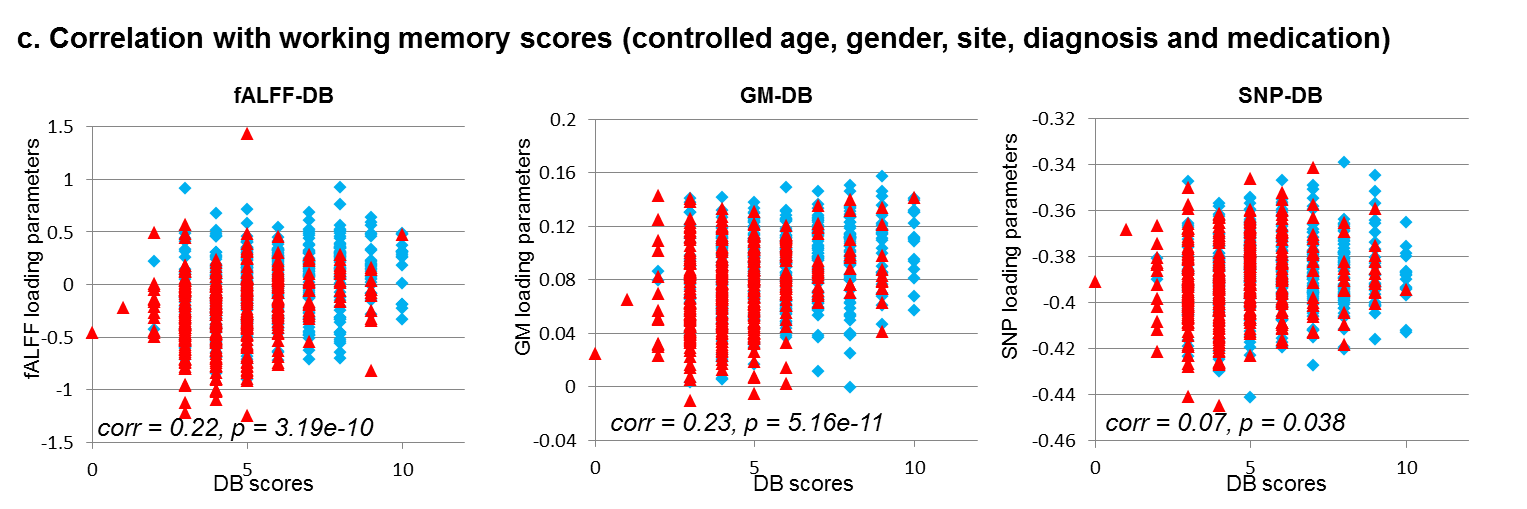


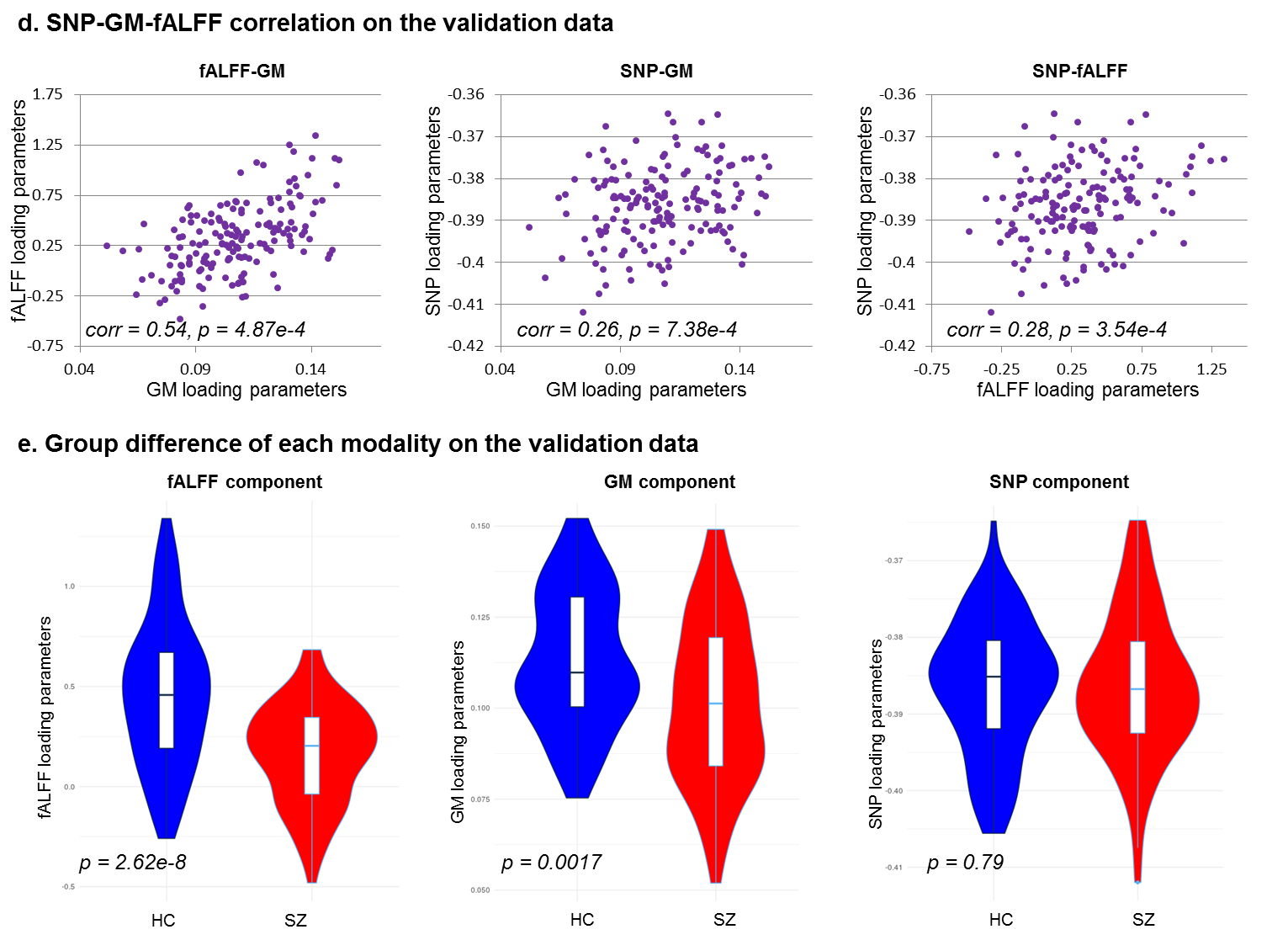


**Fig.S5 Three-way parallel ICA analysis with unregressing age and gender on the SNP modality**


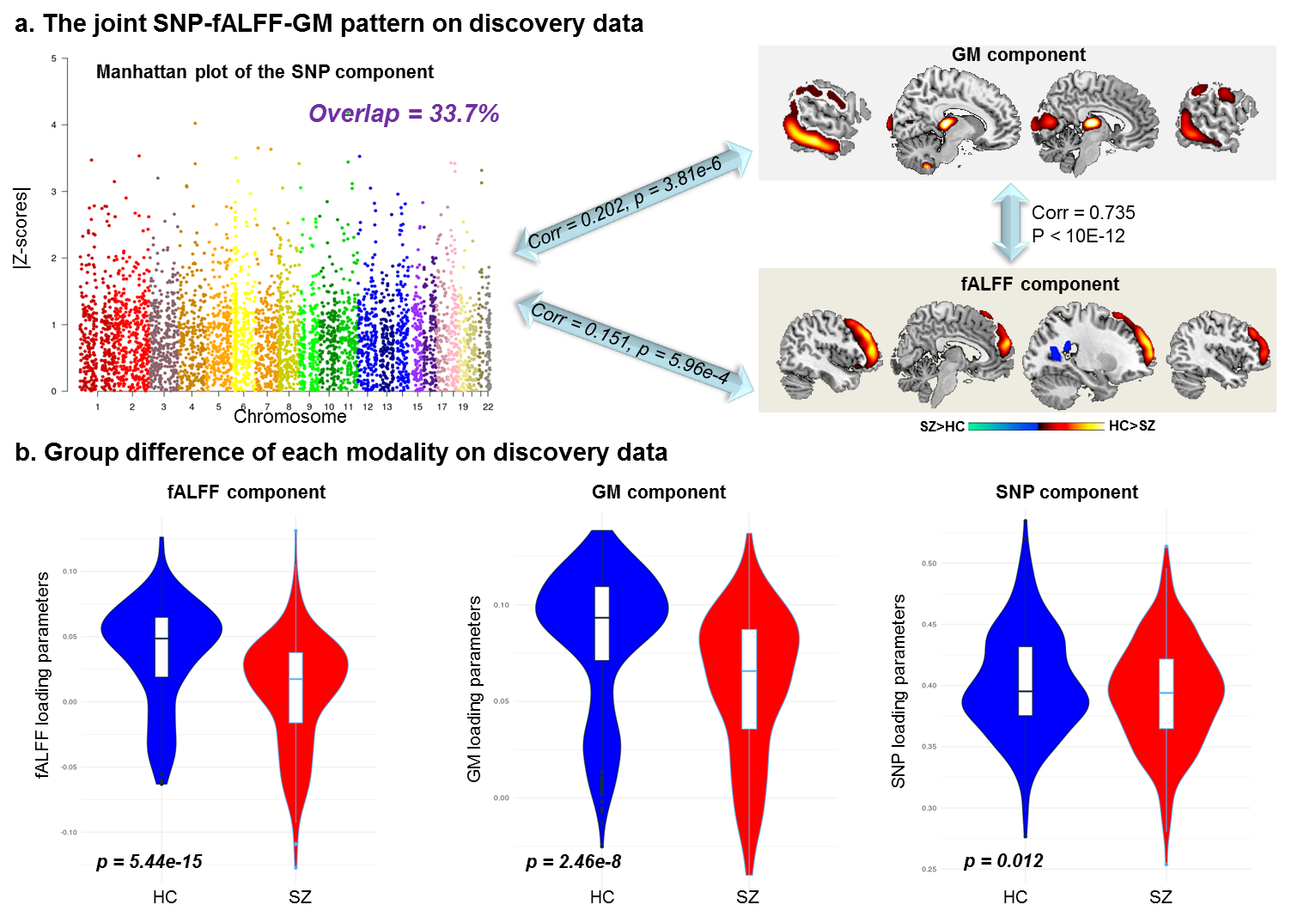


##### **Fig.S6 The three-way para-ICA analysis on the medication dataset**


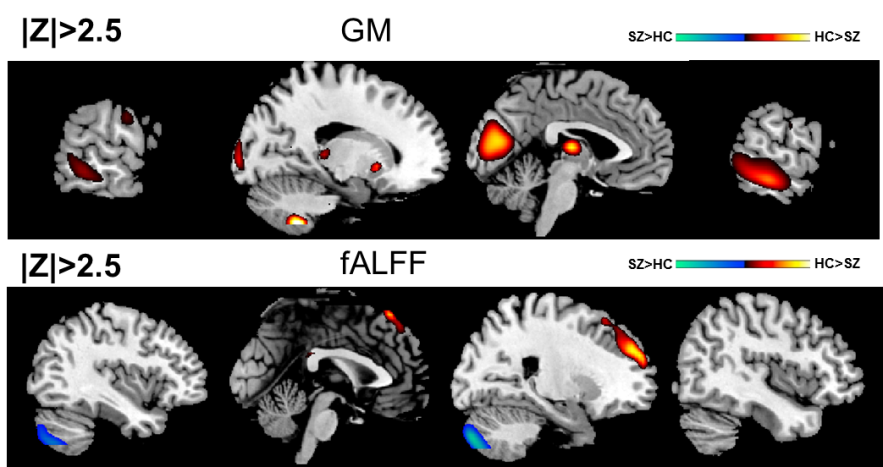


##### **Fig.S7 The spatial maps visualize at |Z|>2.5 for the imaging components**


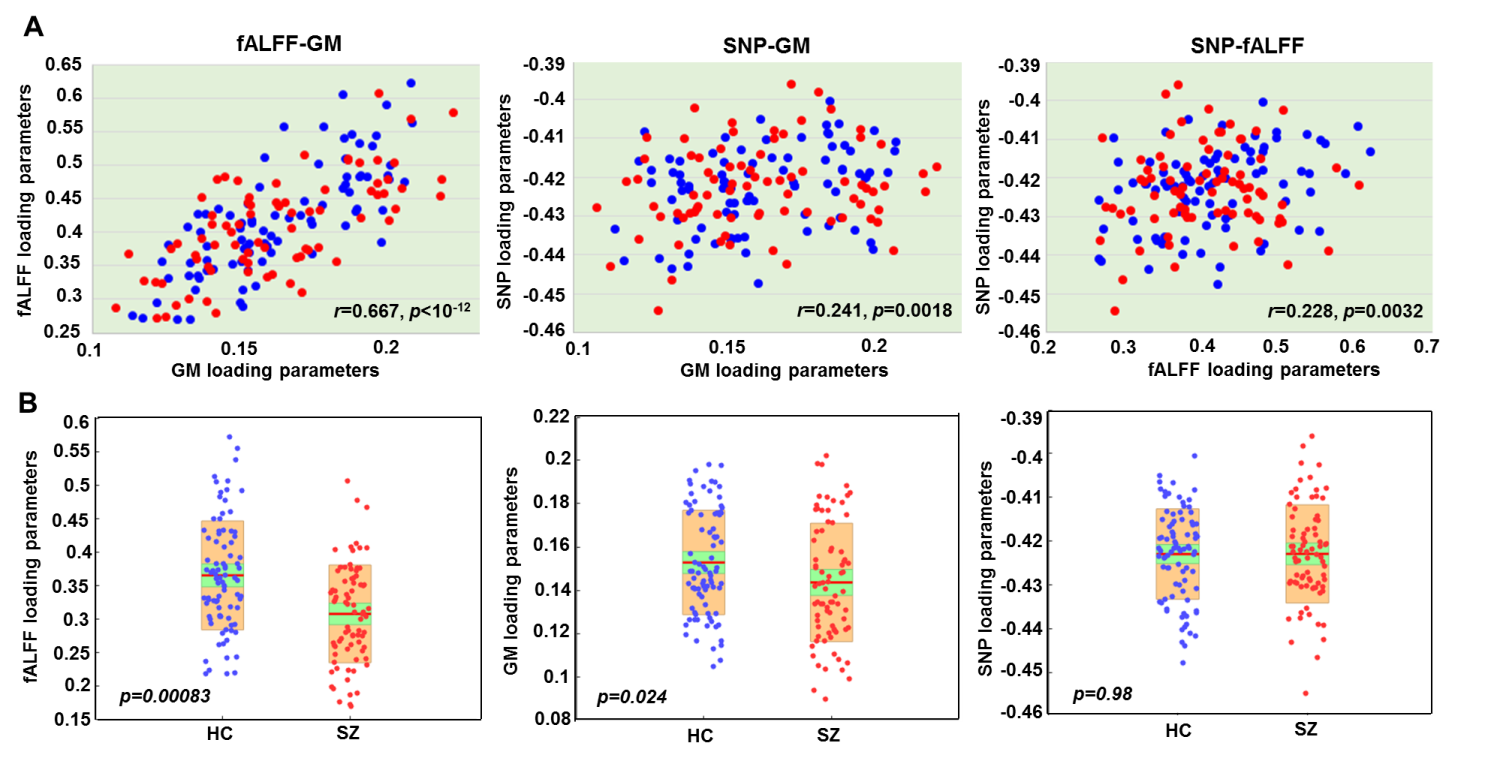


##### **Fig.S8 The projected loadings of the identified joint fALFF-GM-SNP components in the validation dataset.** A. Scatterplots of the partial correlation among the projected fALFF-GM-SNP pattern of SZ (red dot) and HC (blue dot) in the validation dataset. B: loading plots of pairwise t test between SZ and HC for the projected loadings in the validation dataset.

| 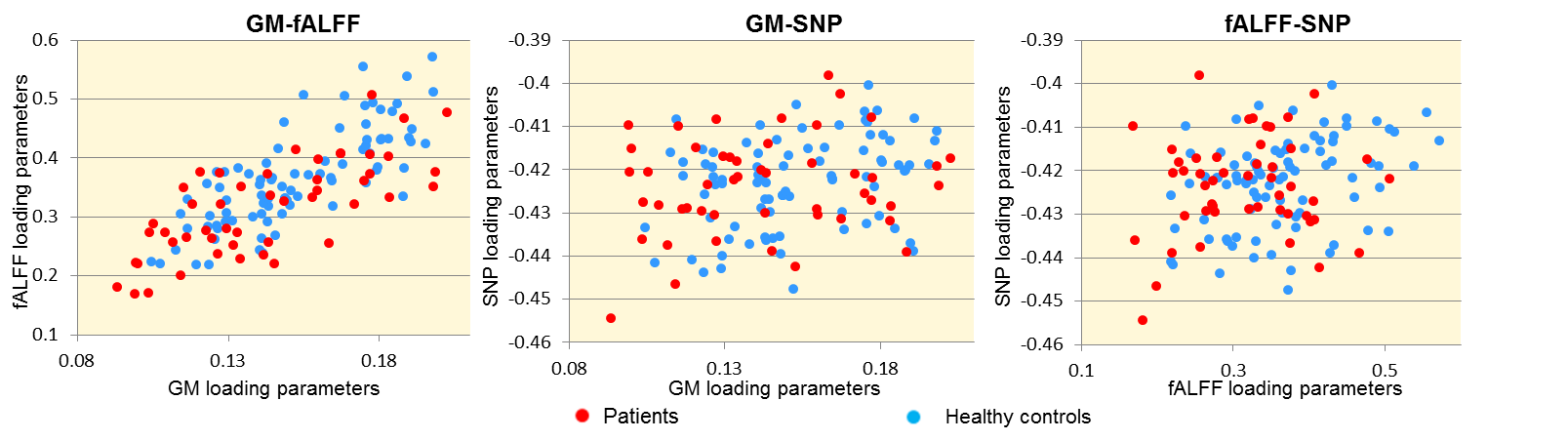 |
| --- |

##### **Fig.S9 Correlation of the projected loadings in the subset of cohort 2**
